# Preferences for COVID-19 vaccine distribution strategies in the US: A discrete choice survey

**DOI:** 10.1371/journal.pone.0256394

**Published:** 2021-08-20

**Authors:** Ingrid Eshun-Wilson, Aaloke Mody, Khai Hoan Tram, Cory Bradley, Alexander Sheve, Branson Fox, Vetta Thompson, Elvin H. Geng

**Affiliations:** 1 School of Medicine, Division of Infectious Diseases, Washington University, St. Louis, Missouri, United States of America; 2 Brown School at Washington University, St. Louis, Missouri, United States of America; 3 Center for Dissemination & Implementation Research Washington University, St. Louis, Missouri, United States of America; University of Haifa, ISRAEL

## Abstract

**Background:**

The COVID-19 vaccination campaign in the US has been immensely successful in vaccinating those who are receptive, further increases in vaccination rates however will require more innovative approaches to reach those who remain hesitant. Developing vaccination strategies that are modelled on what people want could further increase uptake.

**Methods and findings:**

To inform COVID-19 vaccine distribution strategies that are aligned with public preferences we conducted a discrete choice experiment among the US public (N = 2,895) between March 15 to March 22, 2021. We applied sampling weights, evaluated mean preferences using mixed logit models, and identified latent class preference subgroups. On average, the public prioritized ease, preferring single to two dose vaccinations (mean preference: -0.29; 95%CI: -0.37 to -0.20), vaccinating once rather than annually (mean preference: -0.79; 95%CI: -0.89 to -0.70) and reducing waiting times at vaccination sites. Vaccine enforcement reduced overall vaccine acceptance (mean preference -0.20; 95%CI: -0.30 to -0.10), with a trend of increasing resistance to enforcement with increasing vaccine hesitancy. Latent class analysis identified four distinct preference phenotypes: the first prioritized inherent “vaccine features” (46.1%), the second were concerned about vaccine “service delivery” (8.8%), a third group desired “social proof” of vaccine safety and were susceptible to enforcement (13.2%), and the fourth group were “indifferent” to vaccine and service delivery features and resisted enforcement (31.9%).

**Conclusions:**

This study identifies several critical insights for the COVID-19 public health response. First, identifying preference segments is essential to ensure that vaccination services meet the needs of diverse population subgroups. Second, making vaccination easy and promoting autonomy by simplifying services and offering the public choices (where feasible) may increase uptake in those who remain deliberative. And, third vaccine mandates have the potential to increase vaccination rates in susceptible groups but may simultaneously promote control aversion and resistance in those who are most hesitant.

## Introduction

Even though the rapid development of the COVID-19 vaccine has been heralded as a scientific victory, the United States must confront widespread skepticism, hesitancy or indifference from a substantial proportion of the population. This resistance mirrors a wider crisis in the utilization of efficacious biomedical research, as well as the particularities of the COVID-19 epidemic and exploitation of existing social fissures. Some in the public are skeptical of the safety of a vaccine developed rapidly. Others are unconvinced that COVID-19 represents a serious health problem. Finally, COVID-19 has been uniquely politicized, and membership in a social group or political persuasion intersect with vaccine reluctance and rejection. Success in public health, now more than ever before, depends on more than development of efficacious interventions. Instead, we must understand the perspectives, needs, desires and preferences of the public and design strategies to meet those needs [1].

Although much research focuses on acceptability of the vaccine, strategies to improve vaccination would benefit from more nuanced insights into preferences around both vaccine characteristics and delivery approaches. In rational utility theory—a fundamental theory of behavior in economics—people seek to optimize satisfaction (or happiness) through selecting the optimal mix of goods and services available within particular cost constraints. A preference-based framework reframes the decision to vaccinate, therefore, not as a simple question of acceptability, but rather as the consequence of different attributes of a vaccine and the manner in which it is delivered. This information can be used to inform design of strategies, but many of these characteristics are not empirically known. For example, some advocate for vaccine passports that allow access to desired places (e.g., travel) [2], but others warn that mandates and restrictions can provoke a backlash [3, 4]. In addition, different groups may have different preference profiles that demand segmented vaccination strategies.

Extending work that has characterized the desirability of vaccine safety and efficacy, we use a discrete choice experiment (DCE) to assess desirability of different elements of delivery services [5]. We report on a nationally representative DCE on features of vaccination campaigns for the COVID-19 pandemic to provide information on where, how, when and with what inducements vaccines might best be offered, to inform the design of vaccine program and policy.

## Materials and methods

By offering a respondent a choice between repeated versions of a good or service composed of different characteristics within a set of attributes (product or service features), a DCE can reveal what elements of a good or services are preferred, how strong that preference is (in relation to another), as well the size and characteristics of sub-groups in a population with shared preferences. Discrete choice experiments have been a mainstay of marketing research for decades, and have recently become more widely used in public health to discern the voice of the “consumer” [6].

We followed the ISPOR guidelines for design of choice experiments [6, 7]. The study was approved by the Washington University in St Louis institutional review board.

### Choice experiment attribute and attribute level selection

We explored the literature and followed attribute selection guidelines to first generate a comprehensive list of attributes based on expert interviews, discussion and literature, then conduct data reduction, removal of inappropriate attributes and wording refinement [8, 9]. We then developed a final set of salient, plausible, manipulable, non-dominant attributes that represented as complete as possible a list of vaccination campaign features (Table 1). We specifically excluded vaccine safety and efficacy and set the vaccine at 90% effective with a low adverse event rate in the DCE introduction (in order to fully explore preferences for service delivery and other vaccine features).

**Table 1:** Choice experiment attributes and attribute levels.

Attributes:	Attribute levels:
**Vaccination location**	Local pharmacy (e.g., Walgreens, CVS, other)
	Health service (e.g., hospital, doctor office, community center)
	Local pop-up vaccination service (e.g., fire station, community center, school, workplace)
	At your home (door-to-door vaccination)
	Large vaccination site supported by National Guard (e.g., convention center, stadium)
**Waiting time at vaccination site**	Immediate service
	1 hours
	2 hours
**Vaccination appointment scheduling**	Online website
	Phone Call
	No scheduling required (drop-in appointment)
**Number of doses required per vaccination episode**	1 dose only
	2 doses one month apart
**Vaccination enforcement**	Vaccination is completely voluntary (not enforced in any way)
	Vaccination is required for air travel (local and foreign)
	Vaccination is required to attend work/school
	Vaccination is required to attend public recreational spaces, e.g., movies, concerts, conferences)
**Who has already received the vaccine in your community?**	No one I know
	A few people I know
	Almost everyone I know
**Vaccine frequency**	One vaccination required for life-long protection against severe COVID-19 infection
	Annual vaccinations required to maintain protection against severe COVID-19 infection

### Choice experiment design

We limited the number of attributes in the DCE according to design guidelines (seven attributes) and selected those attributes which we determined to be key decision drivers and of the greatest public health policy significance during the time period. To balance statistical and response efficiency (avoid fatigue in respondents) we opted for 10 questions and two scenarios, and limited the number of attribute levels and prohibited attribute level combinations. We manually removed combinations considered non sensical. To prevent forced response we included a third opt-out (choose neither) scenario. The attribute levels were randomly ordered across participants and each participant received one of 300 versions of the choice experiment. We generated a near-orthogonal (each pair of attribute levels appears equally across the experiment) and near-balanced (each level appears equally often across the experiment), fractional factorial design. We assumed no interactions between attributes. The choice tasks were randomly ordered between participants to prevent bias induced by question order. To later assess internal validity and comprehension of the experiment we included a within-set dominant pair [10]. We used the logit efficiency test to estimate the appropriate sample sizes against simulated utility estimates to ensure utility standard errors remained <0.05, resulting in required a minimum sample of 600 participants for main preference estimation. The survey was designed in Lighthouse studio, Sawtooth software [11].

### Data collection and measurements

We collected data on demographic characteristics including: age, gender, race group, employment status, political affiliation and COVID-19 vaccination status, and intention to vaccinate based on questions from the US Census pulse survey [12]. The survey tool and DCE tool can be found in the S1 Appendix. We recruited participants through Qualtrics Research Services, a recognized leader in survey recruitment methodology, using multiple consumer panels to obtain nationally representative survey samples [13]. We used this approach to ensure both a nationally representative sample and by oversampling particular groups to allow for later choice experiment subgroup analyses. Participants were recruited online and offered incentives which varied by participant preference (e.g. cash, gift cards, airmiles). To improve comprehension of the DCE an example of a simplistic choice experiment was presented to participants prior to eliciting responses to the 10 choice tasks. The survey was piloted in 100 anonymous participants.

### Analyses

We first generated population inverse probability sampling weights to reweight our sample to reflect the US Census population estimates for age, race, gender and vaccination status or intention–(Census Pulse Survey 27: March17—March 29, 2021) (S1 Table) [12]. Mixed logit models were used to generate mean utilities (mean preferences) for the population and standard deviations of the random coefficients [14]. Details of the coding and modelling approach are presented in S2 Appendix. Mixed logit coefficients can be interpreted as the strength of the relative preference for the particular attribute comparison, with positive coefficients representing positive preferences (desirable) and negative coefficients representing negative preferences (less desirable).

We fit up to seven latent class conditional logit models using maximum likelihood estimation of datasets expanded by sampling weights and selected the model with the smallest model fit criterion (Akaike and Bayesian information criterion), the highest mean probability of group membership and the smallest number of participants with a low probability of group membership in each group. We additionally qualitatively evaluated latent classes. We validated latent class membership using cross-validation techniques [15]. We used multinomial logistic regression models and relative risk ratios with 95% confidence intervals to evaluate predictors of latent class membership firstly for participant demographic characteristics and secondly for vaccination status or intention and additionally present marginal probabilities of latent class membership based on these models. Stata version 16.1 statistical software was used for all analyses.

## Results

### Participant characteristics

**Fig 1:**
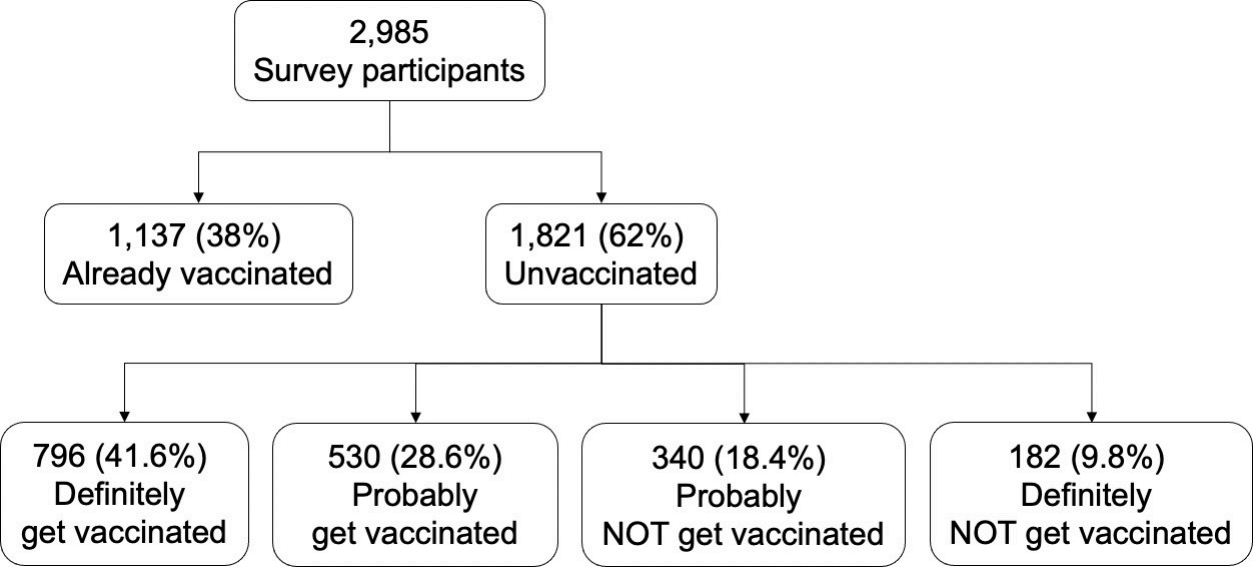
Survey flow diagram for analysis sample. Numbers are unweighted. Survey was delivered between March 15 to March 22, 2021.

The survey was fielded between March 15, 2021 and March 22, 2021 (Fig 1), participant characteristics are presented in Table 2. Of 2,985 survey respondents 38% had already received at least one vaccine dose. Of those who were unvaccinated (1,821 respondents), 41.6% reported that they would definitely get vaccinated, 28.6% would probably get vaccinated, 18.4% would probably NOT get vaccinated and 9.8% would definitely NOT get vaccinated. Participants were located in all 50 US states, as well as the District of Columbia, with 21.7% in the Midwest, 20.4% in the Northeast, 40.1% in the South and 17.8% in the West, 89.0% resided in an urban setting and 11.0% in rural areas. Men made up 37.2% of the sample, with the remaining reporting female gender. The median age was 50 years with an interquartile range (IQR) of 33 to 62 years. 23.5% of the sample was Black/African American, 64.1% White, 7.9% Asian and 4.5% other race groups, and 14.8% identified as Hispanic/Latina.

**Table 2:** Characteristics and COVID experiences of participants.

Participant characteristics		Total (N = 2,985)	Already vaccinated (N = 1,137)	Definitely get a vaccine (N = 796)	Probably get a vaccine (N = 530)	Probably NOT get a vaccine (N = 340)	Definitely NOT get a vaccine (N = 182)
**Age (years)–median; IQR**		50.0 (33.0–62.0)	60.0 (41.0–68.0)	46.0 (34.0–58.0)	41.0 (26.0–55.0)	43.0 (27.0–56.0)	37.5 (22.0–55.0)
**Male gender**		1,111 (37.2%)	448 (39.4%)	329 (41.3%)	175 (33.0%)	101 (29.7%)	58 (31.9%)
**Hispanic/Latino ethnicity**		443 (14.8%)	142 (12.5%)	122 (15.3%)	95 (17.9%)	52 (15.3%)	32 (17.6%)
**Race**	Black/African American	225 (19.8%)	702 (23.5%)	155 (19.5%)	156 (29.4%)	107 (31.5%)	59 (32.4%)
	White	808 (71.1%)	1,913 (64.1%)	508 (63.8%)	299 (56.4%)	191 (56.2%)	107 (58.8%)
	Asian	65 (5.7%)	235 (7.9%)	99 (12.4%)	47 (8.9%)	18 (5.3%)	6 (3.3%)
	Other*	135 (4.5%)	39 (3.4%)	34 (4.3%)	28 (5.3%)	24 (7.1%)	10 (5.5%)
**Schooling**	High school or less	189 (16.6%)	712 (23.9%)	180 (22.6%)	158 (29.8%)	109 (32.1%)	76 (41.8%)
	Incomplete college/Associate degree	354 (31.1%)	993 (33.3%)	252 (31.7%)	187 (35.3%)	136 (40.0%)	64 (35.2%)
	Bachelor degree or higher	594 (52.2%)	1,280 (42.9%)	364 (45.7%)	185 (34.9%)	95 (27.9%)	42 (23.1%)
**Political affiliation**	Democrat	605 (53.2%)	1,425 (47.7%)	439 (55.2%)	216 (40.8%)	103 (30.3%)	62 (34.1%)
	Republican	291 (25.6%)	748 (25.1%)	155 (19.5%)	137 (25.8%)	111 (32.6%)	54 (29.7%)
	Other	67 (5.9%)	222 (7.4%)	48 (6.0%)	58 (10.9%)	35 (10.3%)	14 (7.7%)
	Unaffiliated	156 (13.7%)	476 (15.9%)	128 (16.1%)	85 (16.0%)	70 (20.6%)	37 (20.3%)
	Prefer not to answer	18 (1.6%)	114 (3.8%)	26 (3.3%)	34 (6.4%)	21 (6.2%)	15 (8.2%)
**Region**	Midwest	647 (21.7%)	166 (20.9%)	114 (21.5%)	81 (23.8%)	37 (20.3%)	249 (21.9%)
	Northeast	610 (20.4%)	200 (25.2%)	98 (18.5%)	59 (17.4%)	25 (13.7%)	228 (20.1%)
	South	1,196 (40.1%)	272 (34.2%)	236 (44.5%)	142 (41.8%)	92 (50.5%)	454 (39.9%)
	West	531 (17.8%)	157 (19.7%)	82 (15.5%)	58 (17.1%)	28 (15.4%)	206 (18.1%)
**Setting**	Urban	1,020 (89.7%)	2,656 (89.0%)	700 (88.1%)	482 (90.9%)	290 (85.3%)	164 (90.1%)
	Rural	117 (10.3%)	328 (11.0%)	95 (11.9%)	48 (9.1%)	50 (14.7%)	18 (9.9%)
**Worked for pay/profit in the last 7 days**		1,457 (48.8%)	566 (49.8%)	388 (48.7%)	256 (48.3%)	168 (49.4%)	79 (43.4%)
**Reason for not working in the last 7 days**	Retired or do not want to be employed	732 (47.9%)	412 (72.2%)	151 (37.0%)	80 (29.2%)	54 (31.4%)	35 (34.0%)
	Sick or caring for others -COVID related	109 (7.1%)	17 (3.0%)	33 (8.1%)	31 (11.3%)	19 (11.0%)	9 (8.7%)
	Sick or caring for others—not COVID related	170 (11.1%)	46 (8.1%)	44 (10.8%)	44 (16.1%)	24 (14.0%)	12 (11.7%)
	I was concerned about getting or spreading the coronavirus	74 (4.8%)	15 (2.6%)	35 (8.6%)	15 (5.5%)	6 (3.5%)	3 (2.9%)
	Job loss due to COVID economic impact	176 (11.5%)	35 (6.1%)	63 (15.4%)	33 (12.0%)	32 (18.6%)	13 (12.6%)
	Other reason	267 (17.5%)	46 (8.1%)	82 (20.1%)	71 (25.9%)	37 (21.5%)	31 (30.1%)
**Anyone in the family lost income since Mar13, 2020**		1,117 (37.4%)	391 (34.4%)	309 (38.8%)	220 (41.5%)	133 (39.1%)	64 (35.2%)
**Eligible to receive a COVID-19 vaccine?**	Yes	1,716 (57.5%)	1,137 (100.0%)	261 (32.8%)	158 (29.8%)	100 (29.4%)	60 (33.0%)
	No	928 (31.1%)	0 (0.0%)	456 (57.3%)	258 (48.7%)	137 (40.3%)	77 (42.3%)
	Unsure	341 (11.4%)	0 (0.0%)	79 (9.9%)	114 (21.5%)	103 (30.3%)	45 (24.7%)
**Reason for not vaccinating yet, among eligible who intend to vaccinate**	I tried but could not get an appointment			118 (45.2%)	46 (29.1%)		
	The vaccination site is too far away			18 (6.9%)	21 (13.3%)		
	I could not find information on where to get a vaccination			25 (9.6%)	23 (14.6%)		
	Other reason			100 (38.3%)	68 (43.0%)		
Reasons for hesitancy among those probably/definitely not going to vaccinate**	Deliberative	407 (78%)				278 (81.8%)	129 (70.9%)
	Dissenting	103 (19.7%)				63 (18.5%)	40 (22.0%)
	Distrustful	198 (37.9%)				112 (32.9%)	86 (47.3%)

*Other race includes: American Indian (N = 50); Native Hawaiian (N = 8); Filipino (N = 39); Samoan (N = 1) and other Pacific Islander (N = 37)

**Characterized by Tram et. al [16]. Deliberative reasons: I am concerned about the cost of the vaccine; I think other people need it more; I plan to wait and see if it is safe; My doctor has not recommended it; I don’t know if the vaccine will work; I am concerned about possible side effects; I already had COVID-19; I am not a member of a high-risk group; I plan to use masks or other precautions instead. Distrustful reasons: I don’t trust the government; I don’t trust COVID-19 vaccines.Dissenting reasons: I don’t like vaccines; I don’t believe COVID-19 is a serious illness; I don’t think vaccines are beneficial [16].

Those who probably or definitely did NOT intend to vaccinate most commonly reported deliberative reasons, such as concerns about side-effects and efficacy (78.0%), followed by distrustful reasons including mistrust of the government or COVID vaccines (37.9%), and lastly dissenting reasons such as a lack of belief in vaccines or that COVID-19 is a serious illness (19.7%) [16].

### Main preferences

**Fig 2:**
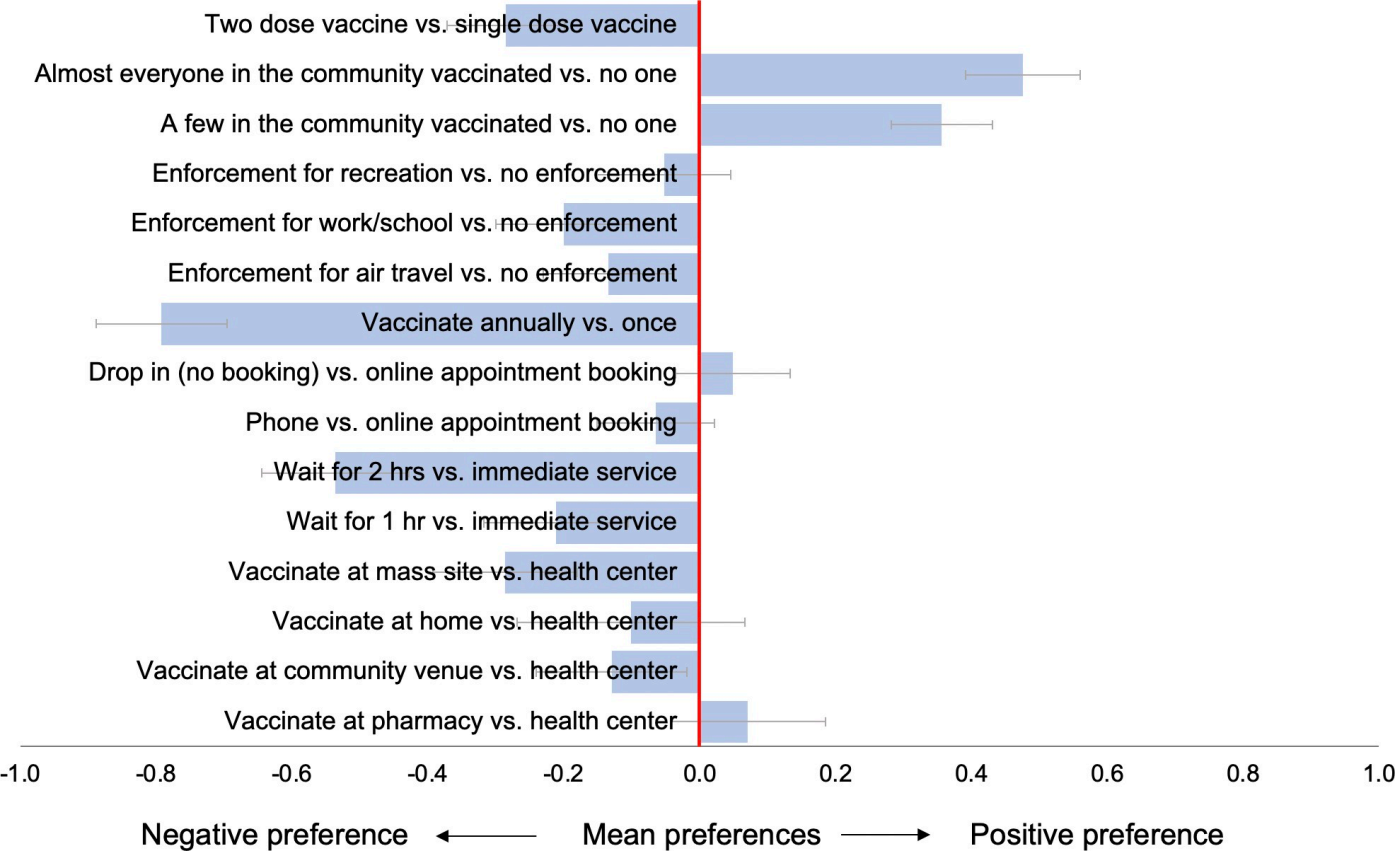
Weighted mean preferences (relative utilities) for vaccination campaign features, in total population (N = 2,985).

In the entire population (N = 2,985), weighted preference estimates for COVID-19 vaccination campaign features are presented in Fig 2 (S2 Table). The strongest preference across attributes was a negative preference for annual (booster) vaccinations compared to one vaccination episode for long-term immunity (mean preference 0.79; 95%CI: -0.89 to -0.70). The US public also preferred to vaccinate at health facilities rather than community venues (mean preference -0.13; 95%CI: -0.24 to -0.02) or mass vaccination sites supported by national guard (mean preference: -0.29; 95%CI: -0.39 to -0.18). Immediate service was preferred to longer waiting times (2 hour mean preference -0.54; 95%CI: -0.64 to -0.43). Participants preferred to vaccinate under voluntary conditions rather than enforcement for air travel (mean preference -0.13; 95%CI: -0.23 to -0.04), work or school attendance (mean preference -0.20; 95%CI: -0.30 to -0.10) or group recreational activities (mean preference -0.05; 95%CI: -0.15 to 0.05). Participants preferred vaccination scenarios where a few people or almost everyone (mean preference 0.48; 95%CI: 0.39 to 0.56) in their community were already vaccinated compared to none. Two vaccination doses were less preferred than a single vaccination dose (mean preference -0.29 to -0.37 to -0.20). There was substantial preference heterogeneity, as evidenced by large standard deviations (SD) for several attributes generated by the mixed logit model (S2 Table).

### Preferences by vaccination status and intention

**Fig 3:**
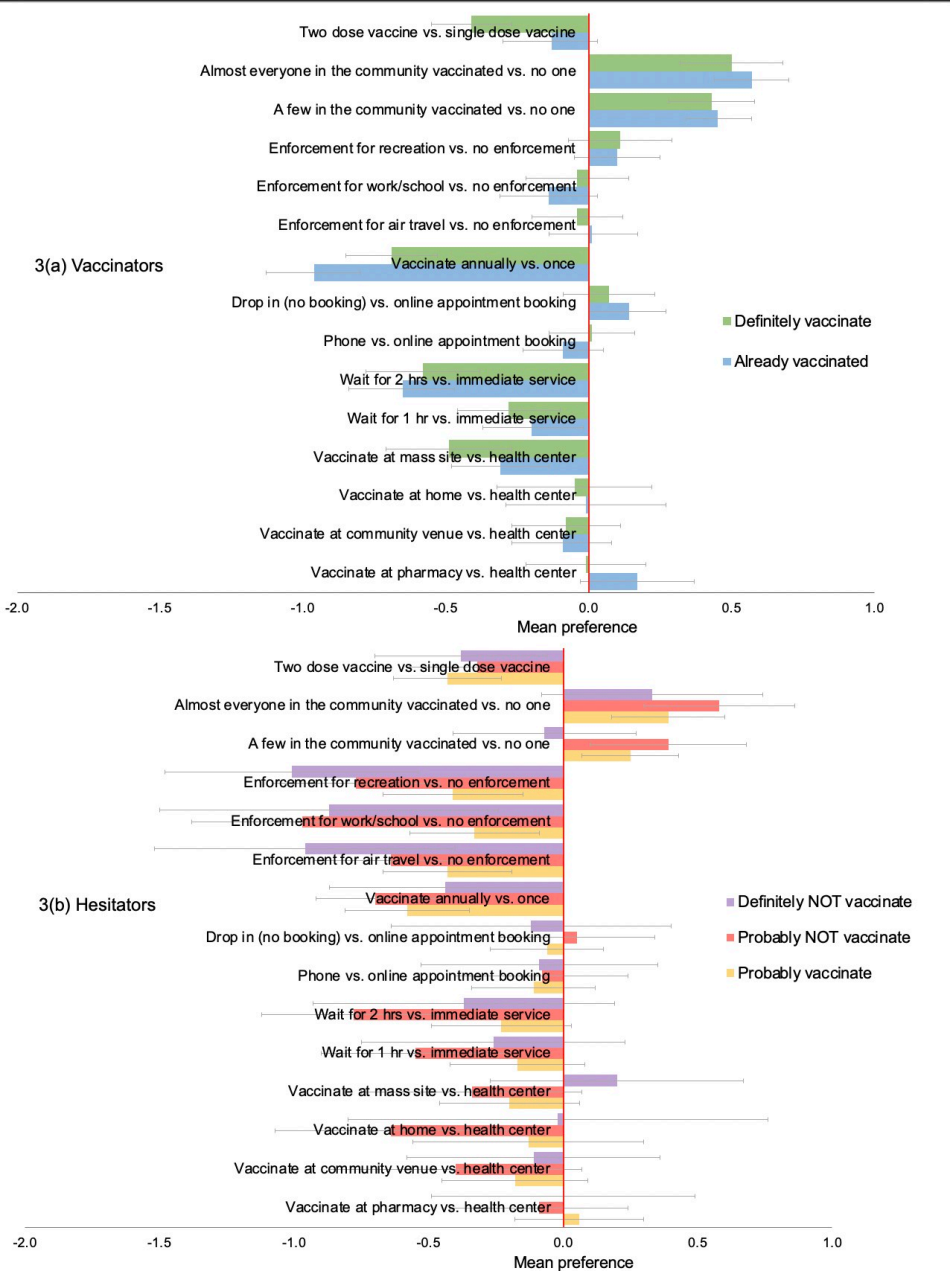
Weighted mean preferences (relative utilities) for vaccination campaign features, among (a) vaccinators and (b) hesitators.

Subgroup analyses by vaccination status and intention showed largely similar preferences among vaccinators (“already vaccinated” or “definitely get vaccinated”) (Fig 3(A), S3 Table) and these mirrored main population preferences. The preferences of the hesitators (“probably get vaccinated”, “probably NOT get vaccinated” or “definitely NOT get vaccinated”) (Fig 3(B), S4 Table), appeared similar to main preferences for waiting time, appointment scheduling, vaccination frequency and number of vaccine doses. But for vaccination enforcement, there was a trend of increasing resistance with increased vaccine hesitancy, those who would “definitely NOT get vaccinated”) were less willing to vaccinate under enforcement (enforcement for work/school vs. no enforcement—mean preference: -0.97; 95%CI: -1.39 to -0.56) compared to those who would “probably get vaccinated” (enforcement for work/school vs. no enforcement—mean preference: -0.33; 95%CI: -0.56 to -0.09). In addition, those who stated they would “definitely NOT vaccinate” were less influenced to vaccinate by expanding vaccination coverage in the community (almost everyone vaccinated vs. no one—mean preference: 0.33; 95%CI: 0.08 to 0.74) compared to less hesitant groups, e.g., those who would “probably NOT get vaccinated” (almost everyone vaccinated vs. no one—mean preference: 0.58; 95%CI: 0.29 to 0.86).

### Latent class analysis

**Fig 4:**
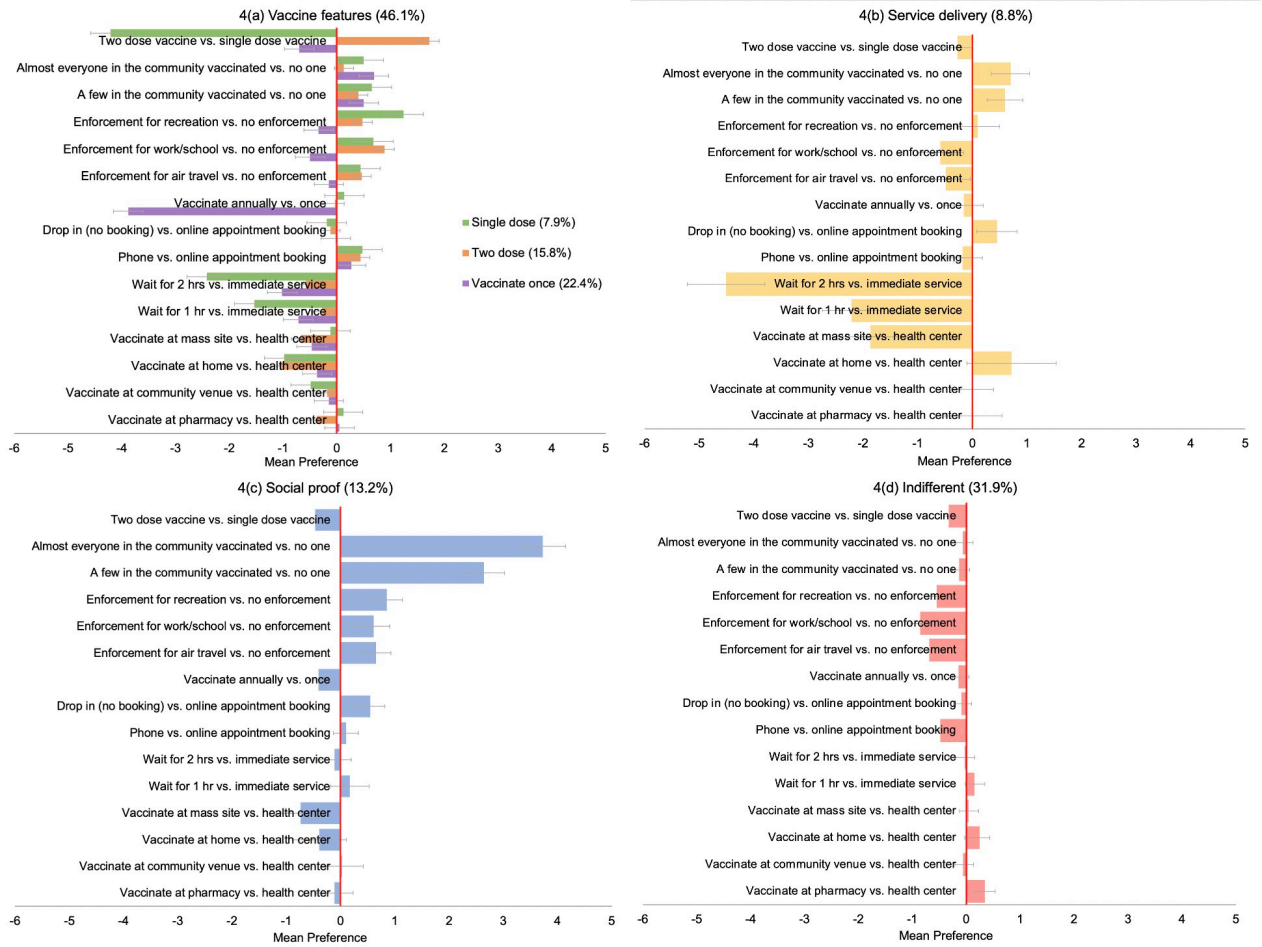
(a-d) Weighted mean preferences (relative utilities) for vaccination campaign features, by latent class membership group.

Latent class analysis revealed six distinct preference subgroups in the population (Fig 4A–4D, S3 Table). Approximately half of the population (46.1%) belonged to one of three preference groups that were concerned about inherent vaccine features such as dosage and frequency (Fig 4A): this included a “single dose” group (7.9%) who showed a strong negative preference for two vaccine doses (mean preference: -4.21; 95%CI: -4.90 to -3.53), a “two dose” group (15.8%) whose strongest preference was for two vaccine doses instead of one (mean preference: 1.72; 95%CI: 1.49 to 1.95) and a “vaccinate once” group (22.4%) whose strongest preference was for a single vaccination episode offering long-term immunity rather than annual vaccination (mean preference: -3.88; 95%CI: -4.18 to -3.57).

The fourth group were focused on vaccine “service delivery”, with strong negative preferences for waiting time (two hours mean preference: -4.51; 95%CI: -5.21 to -3.80) and mass vaccination sites (mean preference: -1.87; 95%CI: -2.55 to -1.19) (Fig 4B).

The fifth preference subgroup, the “social proof” group (13.2%) (Fig 4C) showed a strong positive preference to vaccinate after at least a few or many (mean preference 3.73; 95%CI: 3.31 to 4.15) in their community had already been vaccinated, rather than none. Additionally, they would be more likely to vaccinate if vaccination was enforced (mean preference for work/school enforcement: 0.61; 95%CI: 0.31 to 0.90).

The final preference subgroup, the “indifferent” group (31.9%) (Fig 4D) did not appear to be influenced by inherent vaccine or vaccination service features and showed mild but consistent negative preferences for vaccination enforcement, for work or school (mean preference: -0.86; 95% CI: -1.03 to -0.69), air travel (mean preference: -0.69; 95%CI: -0.86 to -0.52) or recreation (mean preference: -0.55; 95%CI: -0.71 to -0.30).

### Predictors of latent class membership

Age, political affiliation, race and vaccination status/intention were the strongest predictors of latent class membership (Table 3). Compared to membership in the ‘social proof’ preference group, those who were older in age were more likely to prioritize vaccination service features (per 10-yr increased age: RRR 1.16; 95%CI: 1.28 to 3.12) or inherent vaccine features (per 10-yr increased age: RRR 2.00; 95%CI: 1.28 to 3.12). Republicans, were also more likely to vaccinate if their preferred vaccination service delivery approach (RRR 1.73; 95%CI: 1 to 2.99) or inherent vaccine features (RRR 2.36; 95%CI: 1.51 to 3.69) were available, compared to Democrats who were more likely to vaccinate with increasing vaccination coverage or under enforcement (social proof group). Republicans (RRR 2.16; 95%CI: 1.38 to 3.37) and Black/African Americans (RRR 2.00; 95%CI: 1.28 to 3.12) were more likely to be ‘indifferent’ to vaccination strategies and oppose enforcement than belong to the social proof group.

**Table 3:** Weighted multinomial logit model: Predictors of latent class membership for “service features”, “vaccine features” and “indifferent” preference groups relative to the “social proof” preference group (13.2%).

**Participant characteristics**		**Service delivery (8.8%)**				**Vaccine features (46.1%)**				**Indifferent (31.9%)**			
		**RRR**	**Low CI**	**High CI**	**p-value**	**RRR**	**Low CI**	**High CI**	**p-value**	**RRR**	**Low CI**	**High CI**	**p-value**
**Female vs. male**		0.86	0.53	1.41	0.562	0.76	0.54	1.08	0.128	0.74	0.52	1.07	0.109
**Age (per 10-year increase)**		1.14	0.99	1.33	0.077	1.18	1.06	1.32	0.002	0.92	0.83	1.03	0.136
**Hispanic vs. non-Hispanic**		0.94	0.43	2.07	0.886	1.32	0.83	2.10	0.244	1.21	0.71	2.05	0.480
**Race**	White	1.00			0.926	1.00			0.137	1.00			0.006
	Black/African American	1.10	0.60	2.04		1.61	1.03	2.51		1.97	1.25	3.10	
	Asian	1.03	0.48	2.23		0.93	0.53	1.65		0.69	0.38	1.27	
	Other	0.61	0.13	2.80		0.77	0.29	2.08		1.36	0.52	3.56	
**Rural vs. urban**		0.77	0.39	1.50	0.442	0.80	0.51	1.27	0.350	0.85	0.52	1.38	0.512
**No college vs. some college**		1.71	0.97	3.02	0.060	1.55	1.03	2.33	0.036	1.31	0.86	2.00	0.204
**Political affiliation**	Democrat	1.00			0.186	1.00			<0.001	1.00			0.007
	Republican	1.69	0.96	2.95		2.31	1.48	3.60		2.13	1.36	3.34	
	Independent/ other	1.27	0.68	2.34		1.20	0.80	1.80		1.21	0.79	1.87	

*The vaccine features group includes those who preferred a single dose vaccine, a two-dose vaccine or to vaccinate once only.

**Fig 5:**
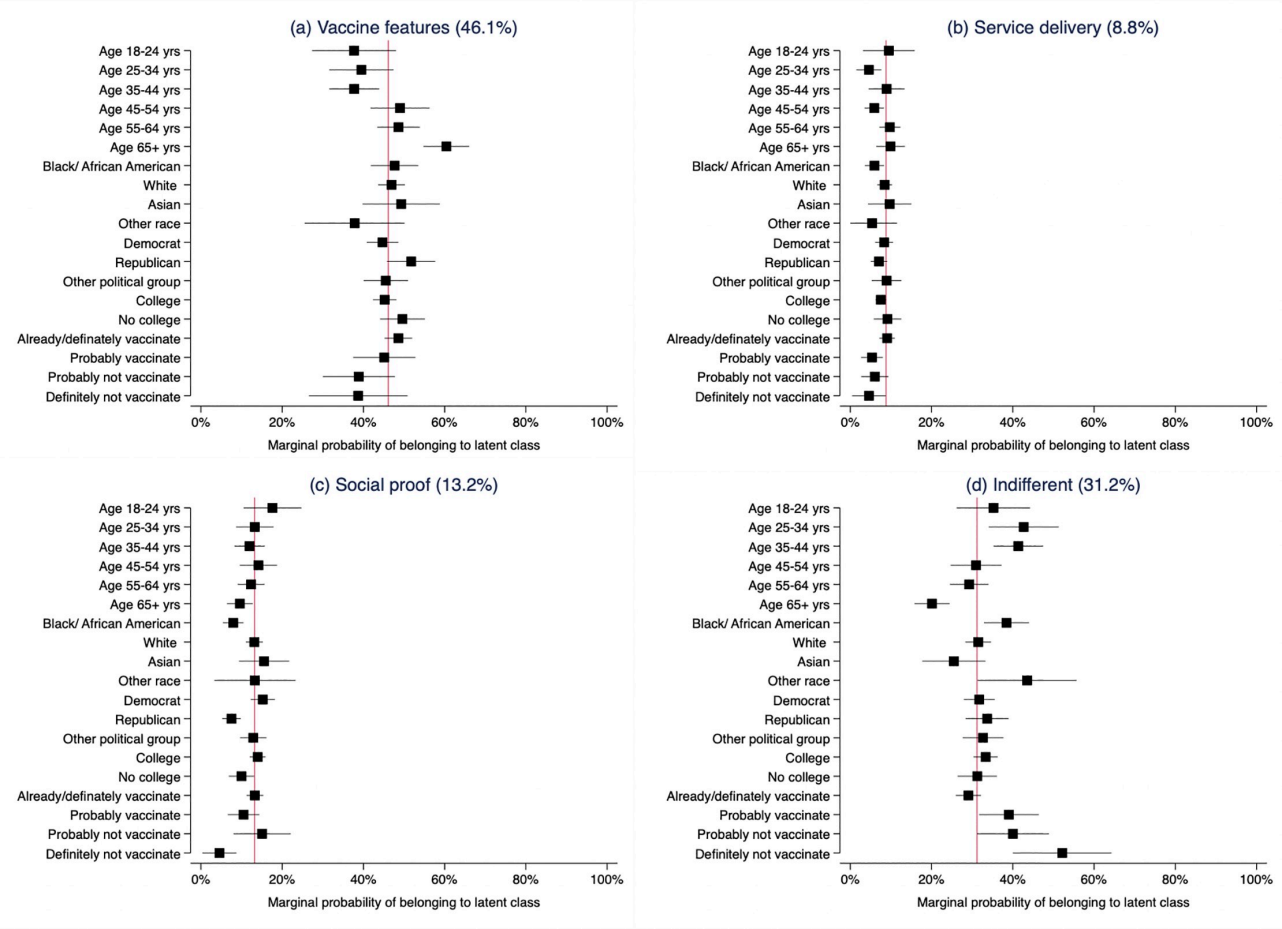
Marginal probability of belonging to latent class group by demographic characteristics and vaccination intention. The red x-line represents overall probability of class membership in the population. “Vaccine features” group includes “single dose”, “two-dose” and “vaccinate once” latent classes.

The marginal probability of belonging to the ‘social proof’ group (Fig 5) was 15.2% for Democrats and 7.5% for Republicans, 7.9% for Black/African Americans, 13.1% for whites and 15.5% for Asians and 17.6% for those 18 to 24 years old compared to 9.6% for those who were over 65 years. The marginal probability of being ‘indifferent’ was 33.7% for Republicans and 31.8% for Democrats, 38.4% for Black/African Americans, 31.5% for whites, 25.5% for Asians and 43.5% for other race groups; 42.7% for those 25–34 years and 20.1% for those over 65 years (Fig 5, S4 Table).

## Discussion

This survey and choice experiment evaluating preferences of the US public for COVID-19 vaccine distribution campaign features demonstrated that US populations prefer to vaccinate under campaigns that provided single rather than multiple vaccine doses, where waiting times at vaccination sites are short and where vaccination is offered at health facilities, pharmacies or in homes and not at mass vaccination sites supported by the national guard. Additionally, the prospect of vaccinating once to get lifelong immunity as opposed to annual vaccination was highly appealing, with the strongest preferences across attributes seen for this potential future vaccine feature. Negative preferences were seen consistently for all vaccination enforcement strategies–air travel, work or school and recreational gatherings, particularly for those who were hesitant. Latent class analyses revealed six preference subgroups in the population, three groups (approximately half the population) prioritized inherent “vaccine features” including dosage and vaccination frequency. A small sub-group (9%), were concerned about vaccination “service delivery” (i.e. waiting time and location). A fifth, “social proof” group (13%) preferred to see others in their community vaccinated before vaccinating themselves and would be more likely to vaccinate under enforcement. A final “indifferent” group (32% of the population) were not influenced by any vaccine or service features, and indicated that they might be less likely to vaccinate under all enforcement strategies. Compared to the social proof group, those who were indifferent were twice as likely to be Republicans rather than Democrats and be Black African/American rather than white or Asian.

These findings firstly provide support for the fundamental behavioral science principle that people are effort minimizers, frequently making health decisions based on the options that are easy and require the least effort [2, 17]. Main preferences for reduced waiting time, single vaccine doses and vaccinating once, highlight the importance of simplifying vaccination processes. And, although early adopters will likely vaccinate under any conditions, for those who continue to be “fence sitters” and deliberate, and in the case where booster vaccinations are required—adjusting services to minimize “friction” in taking up vaccination could help facilitate action [18, 19].

Second, these data suggest that offering a choice of services could increase vaccine uptake. Given, current inequities in global vaccine distribution, the notion of choice remains a luxury afforded by high-income countries but remains relevant to future scenarios where vaccine supply is sufficient and multiple vaccine options are available to all [20]. Choice can promote autonomy and intrinsic motivation, an approach which could be effective in settings such as the US where the group norm is individualism rather than collectivism [21, 22]. In our study over half of the population indicated that they would be more likely to vaccinate under scenarios where their strongly preferred vaccine or vaccination service features were available. Inherent vaccine features regarding safety and efficacy have similarly been shown to be strong preference drivers in previous work [23–27]. Developing simple and efficient systems that allow individuals to take control and choose their preferred vaccine and vaccination delivery service could increase uptake, a strategy which may become more relevant when focus shifts to those who remain unvaccinated despite sufficient supply.

Third, we found that expanding vaccination coverage may provide the social proof of vaccine safety and efficacy needed to increase uptake in those who are deliberative and also susceptible to social influence [1, 28]. The “social proof” preference subgroup—13% of the population—indicated that they would be more likely to take up vaccination with increasing vaccination coverage in the community, and also under enforcement. This group represents those who may be most responsive to normative vaccination campaign messages regarding vaccine safety and coverage, nudges, incentives and vaccine mandates [1].

And finally, these data suggest that vaccine mandates can both increase vaccination rates in those who are susceptible but also promote anti-vaccine sentiment in the most hesitant [4]. Our exploration of preferences for COVID-19 vaccination enforcement strategies including restrictions to air travel, work or school and attendance of recreational gatherings indicated increasing aversion to enforcement with increasing vaccine hesitancy. Such control aversion may operate through several mechanisms, including the impact of the policy message of distrust of the population to be socially responsible, the removal the need to deliberate over vaccination driving out moral convictions to be “prosocial”, inherent mistrust of the government and health systems leading to reduced compliance, and “psychological reactance” [3, 29, 30]. Resistance to vaccination enforcement is not surprising in North American culture which endorses individualism, independence and personal freedom [1, 29, 31]. In addition, affective political polarization is a further critical driver of control aversion in the US setting.

Our findings are strengthened by the use of a representative sample of the US population and the inclusion of survey questions from the US Census Pulse survey allowing for re-weighting to match the US population distribution by age, gender, race, schooling and vaccination status and intention for the corresponding time period of our survey. Results of discrete choice experiments however, particularly in public health, must always be interpreted within a broader body of knowledge and understood within the boundaries of rational choice theory [32]. And although revealed preferences frequently match stated preferences, these assumptions may not always hold in real life decision making, and as a result utilities cannot always perfectly predict uptake of an intervention or service [33]. A further limitation of this study was the use of an online sample which may have influenced preferences for online vaccination appointment scheduling.

Facilitating ease of vaccination and offering a choice of vaccine brands and vaccination services may be best aligned with public preferences for COVID-19 vaccination campaigns features. Several strategies to simplify and incentivize vaccination are already being implemented by various state health departments—our nationally representative data support their use more broadly across the US and highlight the potential challenges with vaccination enforcement strategies in an individualistic society such as the US, where trust in the government is low and where vaccine hesitancy is closely associated with political alliances.

## Supporting information

S1 Table Sampling weights. (DOCX)

S2 Table Main preferences–mixed logit model outputs. (DOCX)

S3 Table Preferences by vaccination status and intention–mixed logit model outputs. (DOCX)

S4 Table Preferences by latent class membership–mixed logit model outputs. (DOCX)

S5 Table Marginal probabilities of latent class group membership. (DOCX)

S1 Appendix Survey tool. (PDF)

S2 Appendix Modelling and coding details. (DOCX)
