# Supplementary material for: Preferences for COVID-19 vaccine distribution strategies in the US: A discrete choice survey
Source: PLoS One. 2021 Aug 20;16(8):e0256394. doi: 10.1371/journal.pone.0256394 (PMC8378751; doi:10.1371/journal.pone.0256394)
Supplement: S1 Table — (DOCX) [file pone.0256394.s001.docx]

| **S1 Table: Pulse Survey population percentages as applied to reweight DCE study sample** | | | | | |
| --- | --- | --- | --- | --- | --- |
| Race | Age Group | Gender | Education | Vaccine Status | Percentage in US population |
| White | 18-24 | Male | High school or less | Definitely get a vaccine | 0.88059670 |
| White | 18-24 | Male | High school or less | Probably get a vaccine | 0.41185856 |
| White | 18-24 | Male | High school or less | Probably NOT get a vaccine | 0.20597842 |
| White | 18-24 | Male | High school or less | Definitely NOT get a vaccine | 0.20889288 |
| White | 18-24 | Male | High school or less | Already vaccinated | 0.14291063 |
| White | 18-24 | Male | Incomplete college/Associate degree | Definitely get a vaccine | 0.65890247 |
| White | 18-24 | Male | Incomplete college/Associate degree | Probably get a vaccine | 0.27298173 |
| White | 18-24 | Male | Incomplete college/Associate degree | Probably NOT get a vaccine | 0.11789024 |
| White | 18-24 | Male | Incomplete college/Associate degree | Definitely NOT get a vaccine | 0.08753720 |
| White | 18-24 | Male | Incomplete college/Associate degree | Already vaccinated | 0.21155982 |
| White | 18-24 | Male | Bachelor degree or higher | Definitely get a vaccine | 0.18038267 |
| White | 18-24 | Male | Bachelor degree or higher | Probably get a vaccine | 0.02283200 |
| White | 18-24 | Male | Bachelor degree or higher | Probably NOT get a vaccine | 0.00799903 |
| White | 18-24 | Male | Bachelor degree or higher | Definitely NOT get a vaccine | 0.01547569 |
| White | 18-24 | Male | Bachelor degree or higher | Already vaccinated | 0.07429212 |
| White | 18-24 | Female | High school or less | Definitely get a vaccine | 0.41138631 |
| White | 18-24 | Female | High school or less | Probably get a vaccine | 0.26984257 |
| White | 18-24 | Female | High school or less | Probably NOT get a vaccine | 0.12144046 |
| White | 18-24 | Female | High school or less | Definitely NOT get a vaccine | 0.10323959 |
| White | 18-24 | Female | High school or less | Already vaccinated | 0.12998548 |
| White | 18-24 | Female | Incomplete college/Associate degree | Definitely get a vaccine | 0.69271505 |
| White | 18-24 | Female | Incomplete college/Associate degree | Probably get a vaccine | 0.17073895 |
| White | 18-24 | Female | Incomplete college/Associate degree | Probably NOT get a vaccine | 0.17620140 |
| White | 18-24 | Female | Incomplete college/Associate degree | Definitely NOT get a vaccine | 0.09844478 |
| White | 18-24 | Female | Incomplete college/Associate degree | Already vaccinated | 0.43343481 |
| White | 18-24 | Female | Bachelor degree or higher | Definitely get a vaccine | 0.12168144 |
| White | 18-24 | Female | Bachelor degree or higher | Probably get a vaccine | 0.03296001 |
| White | 18-24 | Female | Bachelor degree or higher | Probably NOT get a vaccine | 0.03025279 |
| White | 18-24 | Female | Bachelor degree or higher | Definitely NOT get a vaccine | 0.00429185 |
| White | 18-24 | Female | Bachelor degree or higher | Already vaccinated | 0.18734966 |
| White | 25-34 | Male | High school or less | Definitely get a vaccine | 0.55930632 |
| White | 25-34 | Male | High school or less | Probably get a vaccine | 0.43332657 |
| White | 25-34 | Male | High school or less | Probably NOT get a vaccine | 0.37375864 |
| White | 25-34 | Male | High school or less | Definitely NOT get a vaccine | 0.45896083 |
| White | 25-34 | Male | High school or less | Already vaccinated | 0.21719511 |
| White | 25-34 | Male | Incomplete college/Associate degree | Definitely get a vaccine | 0.77293873 |
| White | 25-34 | Male | Incomplete college/Associate degree | Probably get a vaccine | 0.33312801 |
| White | 25-34 | Male | Incomplete college/Associate degree | Probably NOT get a vaccine | 0.18209177 |
| White | 25-34 | Male | Incomplete college/Associate degree | Definitely NOT get a vaccine | 0.23028831 |
| White | 25-34 | Male | Incomplete college/Associate degree | Already vaccinated | 0.58378553 |
| White | 25-34 | Male | Bachelor degree or higher | Definitely get a vaccine | 0.98175013 |
| White | 25-34 | Male | Bachelor degree or higher | Probably get a vaccine | 0.17452127 |
| White | 25-34 | Male | Bachelor degree or higher | Probably NOT get a vaccine | 0.11903349 |
| White | 25-34 | Male | Bachelor degree or higher | Definitely NOT get a vaccine | 0.05970662 |
| White | 25-34 | Male | Bachelor degree or higher | Already vaccinated | 0.84533185 |
| White | 25-34 | Female | High school or less | Definitely get a vaccine | 0.44100893 |
| White | 25-34 | Female | High school or less | Probably get a vaccine | 0.36426613 |
| White | 25-34 | Female | High school or less | Probably NOT get a vaccine | 0.25183254 |
| White | 25-34 | Female | High school or less | Definitely NOT get a vaccine | 0.31789559 |
| White | 25-34 | Female | High school or less | Already vaccinated | 0.34762943 |
| White | 25-34 | Female | Incomplete college/Associate degree | Definitely get a vaccine | 0.63471496 |
| White | 25-34 | Female | Incomplete college/Associate degree | Probably get a vaccine | 0.43889531 |
| White | 25-34 | Female | Incomplete college/Associate degree | Probably NOT get a vaccine | 0.30291691 |
| White | 25-34 | Female | Incomplete college/Associate degree | Definitely NOT get a vaccine | 0.29751211 |
| White | 25-34 | Female | Incomplete college/Associate degree | Already vaccinated | 0.66430008 |
| White | 25-34 | Female | Bachelor degree or higher | Definitely get a vaccine | 0.83233625 |
| White | 25-34 | Female | Bachelor degree or higher | Probably get a vaccine | 0.22707644 |
| White | 25-34 | Female | Bachelor degree or higher | Probably NOT get a vaccine | 0.20688841 |
| White | 25-34 | Female | Bachelor degree or higher | Definitely NOT get a vaccine | 0.09568925 |
| White | 25-34 | Female | Bachelor degree or higher | Already vaccinated | 1.50736952 |
| White | 35-44 | Male | High school or less | Definitely get a vaccine | 0.80106801 |
| White | 35-44 | Male | High school or less | Probably get a vaccine | 0.39562032 |
| White | 35-44 | Male | High school or less | Probably NOT get a vaccine | 0.27381086 |
| White | 35-44 | Male | High school or less | Definitely NOT get a vaccine | 0.37029043 |
| White | 35-44 | Male | High school or less | Already vaccinated | 0.55618668 |
| White | 35-44 | Male | Incomplete college/Associate degree | Definitely get a vaccine | 0.64574122 |
| White | 35-44 | Male | Incomplete college/Associate degree | Probably get a vaccine | 0.25875312 |
| White | 35-44 | Male | Incomplete college/Associate degree | Probably NOT get a vaccine | 0.21732798 |
| White | 35-44 | Male | Incomplete college/Associate degree | Definitely NOT get a vaccine | 0.24663784 |
| White | 35-44 | Male | Incomplete college/Associate degree | Already vaccinated | 0.64785451 |
| White | 35-44 | Male | Bachelor degree or higher | Definitely get a vaccine | 0.72330636 |
| White | 35-44 | Male | Bachelor degree or higher | Probably get a vaccine | 0.17681870 |
| White | 35-44 | Male | Bachelor degree or higher | Probably NOT get a vaccine | 0.11164686 |
| White | 35-44 | Male | Bachelor degree or higher | Definitely NOT get a vaccine | 0.12200039 |
| White | 35-44 | Male | Bachelor degree or higher | Already vaccinated | 1.06056833 |
| White | 35-44 | Female | High school or less | Definitely get a vaccine | 0.47539768 |
| White | 35-44 | Female | High school or less | Probably get a vaccine | 0.46099666 |
| White | 35-44 | Female | High school or less | Probably NOT get a vaccine | 0.24208607 |
| White | 35-44 | Female | High school or less | Definitely NOT get a vaccine | 0.30721790 |
| White | 35-44 | Female | High school or less | Already vaccinated | 0.43671286 |
| White | 35-44 | Female | Incomplete college/Associate degree | Definitely get a vaccine | 0.38280156 |
| White | 35-44 | Female | Incomplete college/Associate degree | Probably get a vaccine | 0.29467374 |
| White | 35-44 | Female | Incomplete college/Associate degree | Probably NOT get a vaccine | 0.25390655 |
| White | 35-44 | Female | Incomplete college/Associate degree | Definitely NOT get a vaccine | 0.24215461 |
| White | 35-44 | Female | Incomplete college/Associate degree | Already vaccinated | 0.63146037 |
| White | 35-44 | Female | Bachelor degree or higher | Definitely get a vaccine | 0.65702331 |
| White | 35-44 | Female | Bachelor degree or higher | Probably get a vaccine | 0.18878050 |
| White | 35-44 | Female | Bachelor degree or higher | Probably NOT get a vaccine | 0.16097838 |
| White | 35-44 | Female | Bachelor degree or higher | Definitely NOT get a vaccine | 0.10673342 |
| White | 35-44 | Female | Bachelor degree or higher | Already vaccinated | 1.43354511 |
| White | 45-54 | Male | High school or less | Definitely get a vaccine | 0.78012407 |
| White | 45-54 | Male | High school or less | Probably get a vaccine | 0.33711344 |
| White | 45-54 | Male | High school or less | Probably NOT get a vaccine | 0.21099958 |
| White | 45-54 | Male | High school or less | Definitely NOT get a vaccine | 0.38228554 |
| White | 45-54 | Male | High school or less | Already vaccinated | 0.73247677 |
| White | 45-54 | Male | Incomplete college/Associate degree | Definitely get a vaccine | 0.42353806 |
| White | 45-54 | Male | Incomplete college/Associate degree | Probably get a vaccine | 0.23593360 |
| White | 45-54 | Male | Incomplete college/Associate degree | Probably NOT get a vaccine | 0.14247265 |
| White | 45-54 | Male | Incomplete college/Associate degree | Definitely NOT get a vaccine | 0.14490201 |
| White | 45-54 | Male | Incomplete college/Associate degree | Already vaccinated | 0.66418236 |
| White | 45-54 | Male | Bachelor degree or higher | Definitely get a vaccine | 0.60467201 |
| White | 45-54 | Male | Bachelor degree or higher | Probably get a vaccine | 0.19385101 |
| White | 45-54 | Male | Bachelor degree or higher | Probably NOT get a vaccine | 0.06460436 |
| White | 45-54 | Male | Bachelor degree or higher | Definitely NOT get a vaccine | 0.07184025 |
| White | 45-54 | Male | Bachelor degree or higher | Already vaccinated | 0.97267097 |
| White | 45-54 | Female | High school or less | Definitely get a vaccine | 0.71404314 |
| White | 45-54 | Female | High school or less | Probably get a vaccine | 0.39102462 |
| White | 45-54 | Female | High school or less | Probably NOT get a vaccine | 0.24584676 |
| White | 45-54 | Female | High school or less | Definitely NOT get a vaccine | 0.24102242 |
| White | 45-54 | Female | High school or less | Already vaccinated | 0.58880097 |
| White | 45-54 | Female | Incomplete college/Associate degree | Definitely get a vaccine | 0.38530064 |
| White | 45-54 | Female | Incomplete college/Associate degree | Probably get a vaccine | 0.22766306 |
| White | 45-54 | Female | Incomplete college/Associate degree | Probably NOT get a vaccine | 0.18935494 |
| White | 45-54 | Female | Incomplete college/Associate degree | Definitely NOT get a vaccine | 0.18744701 |
| White | 45-54 | Female | Incomplete college/Associate degree | Already vaccinated | 0.81366622 |
| White | 45-54 | Female | Bachelor degree or higher | Definitely get a vaccine | 0.48854232 |
| White | 45-54 | Female | Bachelor degree or higher | Probably get a vaccine | 0.16724294 |
| White | 45-54 | Female | Bachelor degree or higher | Probably NOT get a vaccine | 0.11898842 |
| White | 45-54 | Female | Bachelor degree or higher | Definitely NOT get a vaccine | 0.08746195 |
| White | 45-54 | Female | Bachelor degree or higher | Already vaccinated | 1.23043501 |
| White | 55-64 | Male | High school or less | Definitely get a vaccine | 0.73256952 |
| White | 55-64 | Male | High school or less | Probably get a vaccine | 0.40250343 |
| White | 55-64 | Male | High school or less | Probably NOT get a vaccine | 0.23209365 |
| White | 55-64 | Male | High school or less | Definitely NOT get a vaccine | 0.21451372 |
| White | 55-64 | Male | High school or less | Already vaccinated | 1.28672719 |
| White | 55-64 | Male | Incomplete college/Associate degree | Definitely get a vaccine | 0.43482670 |
| White | 55-64 | Male | Incomplete college/Associate degree | Probably get a vaccine | 0.19126257 |
| White | 55-64 | Male | Incomplete college/Associate degree | Probably NOT get a vaccine | 0.11919882 |
| White | 55-64 | Male | Incomplete college/Associate degree | Definitely NOT get a vaccine | 0.15724355 |
| White | 55-64 | Male | Incomplete college/Associate degree | Already vaccinated | 0.94029742 |
| White | 55-64 | Male | Bachelor degree or higher | Definitely get a vaccine | 0.48590744 |
| White | 55-64 | Male | Bachelor degree or higher | Probably get a vaccine | 0.11547807 |
| White | 55-64 | Male | Bachelor degree or higher | Probably NOT get a vaccine | 0.05247556 |
| White | 55-64 | Male | Bachelor degree or higher | Definitely NOT get a vaccine | 0.06422006 |
| White | 55-64 | Male | Bachelor degree or higher | Already vaccinated | 1.12754321 |
| White | 55-64 | Female | High school or less | Definitely get a vaccine | 0.66534728 |
| White | 55-64 | Female | High school or less | Probably get a vaccine | 0.36104518 |
| White | 55-64 | Female | High school or less | Probably NOT get a vaccine | 0.22285943 |
| White | 55-64 | Female | High school or less | Definitely NOT get a vaccine | 0.16690432 |
| White | 55-64 | Female | High school or less | Already vaccinated | 1.28392661 |
| White | 55-64 | Female | Incomplete college/Associate degree | Definitely get a vaccine | 0.47443166 |
| White | 55-64 | Female | Incomplete college/Associate degree | Probably get a vaccine | 0.22045954 |
| White | 55-64 | Female | Incomplete college/Associate degree | Probably NOT get a vaccine | 0.14498445 |
| White | 55-64 | Female | Incomplete college/Associate degree | Definitely NOT get a vaccine | 0.11059872 |
| White | 55-64 | Female | Incomplete college/Associate degree | Already vaccinated | 1.15686619 |
| White | 55-64 | Female | Bachelor degree or higher | Definitely get a vaccine | 0.42633542 |
| White | 55-64 | Female | Bachelor degree or higher | Probably get a vaccine | 0.10102709 |
| White | 55-64 | Female | Bachelor degree or higher | Probably NOT get a vaccine | 0.07827702 |
| White | 55-64 | Female | Bachelor degree or higher | Definitely NOT get a vaccine | 0.05280546 |
| White | 55-64 | Female | Bachelor degree or higher | Already vaccinated | 1.33369315 |
| White | 65+ | Male | High school or less | Definitely get a vaccine | 0.39842489 |
| White | 65+ | Male | High school or less | Probably get a vaccine | 0.14682660 |
| White | 65+ | Male | High school or less | Probably NOT get a vaccine | 0.17538460 |
| White | 65+ | Male | High school or less | Definitely NOT get a vaccine | 0.12438075 |
| White | 65+ | Male | High school or less | Already vaccinated | 2.46911931 |
| White | 65+ | Male | Incomplete college/Associate degree | Definitely get a vaccine | 0.17081732 |
| White | 65+ | Male | Incomplete college/Associate degree | Probably get a vaccine | 0.08464184 |
| White | 65+ | Male | Incomplete college/Associate degree | Probably NOT get a vaccine | 0.05811615 |
| White | 65+ | Male | Incomplete college/Associate degree | Definitely NOT get a vaccine | 0.07587925 |
| White | 65+ | Male | Incomplete college/Associate degree | Already vaccinated | 1.75297391 |
| White | 65+ | Male | Bachelor degree or higher | Definitely get a vaccine | 0.14756335 |
| White | 65+ | Male | Bachelor degree or higher | Probably get a vaccine | 0.05802047 |
| White | 65+ | Male | Bachelor degree or higher | Probably NOT get a vaccine | 0.04194709 |
| White | 65+ | Male | Bachelor degree or higher | Definitely NOT get a vaccine | 0.04490284 |
| White | 65+ | Male | Bachelor degree or higher | Already vaccinated | 2.45612597 |
| White | 65+ | Female | High school or less | Definitely get a vaccine | 0.41485068 |
| White | 65+ | Female | High school or less | Probably get a vaccine | 0.26718476 |
| White | 65+ | Female | High school or less | Probably NOT get a vaccine | 0.21084957 |
| White | 65+ | Female | High school or less | Definitely NOT get a vaccine | 0.16612864 |
| White | 65+ | Female | High school or less | Already vaccinated | 3.68626857 |
| White | 65+ | Female | Incomplete college/Associate degree | Definitely get a vaccine | 0.18964103 |
| White | 65+ | Female | Incomplete college/Associate degree | Probably get a vaccine | 0.10361837 |
| White | 65+ | Female | Incomplete college/Associate degree | Probably NOT get a vaccine | 0.10089190 |
| White | 65+ | Female | Incomplete college/Associate degree | Definitely NOT get a vaccine | 0.10063599 |
| White | 65+ | Female | Incomplete college/Associate degree | Already vaccinated | 2.16050768 |
| White | 65+ | Female | Bachelor degree or higher | Definitely get a vaccine | 0.12978722 |
| White | 65+ | Female | Bachelor degree or higher | Probably get a vaccine | 0.05568712 |
| White | 65+ | Female | Bachelor degree or higher | Probably NOT get a vaccine | 0.04837983 |
| White | 65+ | Female | Bachelor degree or higher | Definitely NOT get a vaccine | 0.04076084 |
| White | 65+ | Female | Bachelor degree or higher | Already vaccinated | 2.12997341 |
| Black | 18-24 | Male | High school or less | Definitely get a vaccine | 0.04254603 |
| Black | 18-24 | Male | High school or less | Probably get a vaccine | 0.06432409 |
| Black | 18-24 | Male | High school or less | Probably NOT get a vaccine | 0.00000000 |
| Black | 18-24 | Male | High school or less | Definitely NOT get a vaccine | 0.03150065 |
| Black | 18-24 | Male | High school or less | Already vaccinated | 0.03623263 |
| Black | 18-24 | Male | Incomplete college/Associate degree | Definitely get a vaccine | 0.04990794 |
| Black | 18-24 | Male | Incomplete college/Associate degree | Probably get a vaccine | 0.03823696 |
| Black | 18-24 | Male | Incomplete college/Associate degree | Probably NOT get a vaccine | 0.04109951 |
| Black | 18-24 | Male | Incomplete college/Associate degree | Definitely NOT get a vaccine | 0.01017068 |
| Black | 18-24 | Male | Incomplete college/Associate degree | Already vaccinated | 0.01273386 |
| Black | 18-24 | Male | Bachelor degree or higher | Definitely get a vaccine | 0.01869441 |
| Black | 18-24 | Male | Bachelor degree or higher | Probably get a vaccine | 0.00000000 |
| Black | 18-24 | Male | Bachelor degree or higher | Probably NOT get a vaccine | 0.00297286 |
| Black | 18-24 | Male | Bachelor degree or higher | Definitely NOT get a vaccine | 0.00000000 |
| Black | 18-24 | Male | Bachelor degree or higher | Already vaccinated | 0.00203632 |
| Black | 18-24 | Female | High school or less | Definitely get a vaccine | 0.04540189 |
| Black | 18-24 | Female | High school or less | Probably get a vaccine | 0.02947550 |
| Black | 18-24 | Female | High school or less | Probably NOT get a vaccine | 0.02006121 |
| Black | 18-24 | Female | High school or less | Definitely NOT get a vaccine | 0.02300096 |
| Black | 18-24 | Female | High school or less | Already vaccinated | 0.00391023 |
| Black | 18-24 | Female | Incomplete college/Associate degree | Definitely get a vaccine | 0.04535841 |
| Black | 18-24 | Female | Incomplete college/Associate degree | Probably get a vaccine | 0.08136075 |
| Black | 18-24 | Female | Incomplete college/Associate degree | Probably NOT get a vaccine | 0.01870689 |
| Black | 18-24 | Female | Incomplete college/Associate degree | Definitely NOT get a vaccine | 0.02017083 |
| Black | 18-24 | Female | Incomplete college/Associate degree | Already vaccinated | 0.05532825 |
| Black | 18-24 | Female | Bachelor degree or higher | Definitely get a vaccine | 0.01165846 |
| Black | 18-24 | Female | Bachelor degree or higher | Probably get a vaccine | 0.01256022 |
| Black | 18-24 | Female | Bachelor degree or higher | Probably NOT get a vaccine | 0.01190885 |
| Black | 18-24 | Female | Bachelor degree or higher | Definitely NOT get a vaccine | 0.00000000 |
| Black | 18-24 | Female | Bachelor degree or higher | Already vaccinated | 0.00819009 |
| Black | 25-34 | Male | High school or less | Definitely get a vaccine | 0.07604944 |
| Black | 25-34 | Male | High school or less | Probably get a vaccine | 0.08116805 |
| Black | 25-34 | Male | High school or less | Probably NOT get a vaccine | 0.04534615 |
| Black | 25-34 | Male | High school or less | Definitely NOT get a vaccine | 0.10170152 |
| Black | 25-34 | Male | High school or less | Already vaccinated | 0.01981156 |
| Black | 25-34 | Male | Incomplete college/Associate degree | Definitely get a vaccine | 0.04979984 |
| Black | 25-34 | Male | Incomplete college/Associate degree | Probably get a vaccine | 0.11900820 |
| Black | 25-34 | Male | Incomplete college/Associate degree | Probably NOT get a vaccine | 0.03680303 |
| Black | 25-34 | Male | Incomplete college/Associate degree | Definitely NOT get a vaccine | 0.00334251 |
| Black | 25-34 | Male | Incomplete college/Associate degree | Already vaccinated | 0.01193117 |
| Black | 25-34 | Male | Bachelor degree or higher | Definitely get a vaccine | 0.06161587 |
| Black | 25-34 | Male | Bachelor degree or higher | Probably get a vaccine | 0.04612500 |
| Black | 25-34 | Male | Bachelor degree or higher | Probably NOT get a vaccine | 0.00854158 |
| Black | 25-34 | Male | Bachelor degree or higher | Definitely NOT get a vaccine | 0.00036173 |
| Black | 25-34 | Male | Bachelor degree or higher | Already vaccinated | 0.06519294 |
| Black | 25-34 | Female | High school or less | Definitely get a vaccine | 0.08240709 |
| Black | 25-34 | Female | High school or less | Probably get a vaccine | 0.11121935 |
| Black | 25-34 | Female | High school or less | Probably NOT get a vaccine | 0.10904006 |
| Black | 25-34 | Female | High school or less | Definitely NOT get a vaccine | 0.13209657 |
| Black | 25-34 | Female | High school or less | Already vaccinated | 0.04426508 |
| Black | 25-34 | Female | Incomplete college/Associate degree | Definitely get a vaccine | 0.08719063 |
| Black | 25-34 | Female | Incomplete college/Associate degree | Probably get a vaccine | 0.09444108 |
| Black | 25-34 | Female | Incomplete college/Associate degree | Probably NOT get a vaccine | 0.10006076 |
| Black | 25-34 | Female | Incomplete college/Associate degree | Definitely NOT get a vaccine | 0.05717136 |
| Black | 25-34 | Female | Incomplete college/Associate degree | Already vaccinated | 0.09763716 |
| Black | 25-34 | Female | Bachelor degree or higher | Definitely get a vaccine | 0.07728636 |
| Black | 25-34 | Female | Bachelor degree or higher | Probably get a vaccine | 0.07221153 |
| Black | 25-34 | Female | Bachelor degree or higher | Probably NOT get a vaccine | 0.03335232 |
| Black | 25-34 | Female | Bachelor degree or higher | Definitely NOT get a vaccine | 0.02093162 |
| Black | 25-34 | Female | Bachelor degree or higher | Already vaccinated | 0.11235956 |
| Black | 35-44 | Male | High school or less | Definitely get a vaccine | 0.18304512 |
| Black | 35-44 | Male | High school or less | Probably get a vaccine | 0.08327250 |
| Black | 35-44 | Male | High school or less | Probably NOT get a vaccine | 0.10193397 |
| Black | 35-44 | Male | High school or less | Definitely NOT get a vaccine | 0.09855625 |
| Black | 35-44 | Male | High school or less | Already vaccinated | 0.08943479 |
| Black | 35-44 | Male | Incomplete college/Associate degree | Definitely get a vaccine | 0.17146583 |
| Black | 35-44 | Male | Incomplete college/Associate degree | Probably get a vaccine | 0.07702290 |
| Black | 35-44 | Male | Incomplete college/Associate degree | Probably NOT get a vaccine | 0.03318253 |
| Black | 35-44 | Male | Incomplete college/Associate degree | Definitely NOT get a vaccine | 0.06048685 |
| Black | 35-44 | Male | Incomplete college/Associate degree | Already vaccinated | 0.12700726 |
| Black | 35-44 | Male | Bachelor degree or higher | Definitely get a vaccine | 0.07688067 |
| Black | 35-44 | Male | Bachelor degree or higher | Probably get a vaccine | 0.03699781 |
| Black | 35-44 | Male | Bachelor degree or higher | Probably NOT get a vaccine | 0.01014439 |
| Black | 35-44 | Male | Bachelor degree or higher | Definitely NOT get a vaccine | 0.00784530 |
| Black | 35-44 | Male | Bachelor degree or higher | Already vaccinated | 0.08971959 |
| Black | 35-44 | Female | High school or less | Definitely get a vaccine | 0.13272803 |
| Black | 35-44 | Female | High school or less | Probably get a vaccine | 0.13793813 |
| Black | 35-44 | Female | High school or less | Probably NOT get a vaccine | 0.14260516 |
| Black | 35-44 | Female | High school or less | Definitely NOT get a vaccine | 0.11364584 |
| Black | 35-44 | Female | High school or less | Already vaccinated | 0.18334116 |
| Black | 35-44 | Female | Incomplete college/Associate degree | Definitely get a vaccine | 0.09592485 |
| Black | 35-44 | Female | Incomplete college/Associate degree | Probably get a vaccine | 0.08537077 |
| Black | 35-44 | Female | Incomplete college/Associate degree | Probably NOT get a vaccine | 0.11339032 |
| Black | 35-44 | Female | Incomplete college/Associate degree | Definitely NOT get a vaccine | 0.06999999 |
| Black | 35-44 | Female | Incomplete college/Associate degree | Already vaccinated | 0.12403256 |
| Black | 35-44 | Female | Bachelor degree or higher | Definitely get a vaccine | 0.06652909 |
| Black | 35-44 | Female | Bachelor degree or higher | Probably get a vaccine | 0.07837132 |
| Black | 35-44 | Female | Bachelor degree or higher | Probably NOT get a vaccine | 0.06699082 |
| Black | 35-44 | Female | Bachelor degree or higher | Definitely NOT get a vaccine | 0.01266304 |
| Black | 35-44 | Female | Bachelor degree or higher | Already vaccinated | 0.18936825 |
| Black | 45-54 | Male | High school or less | Definitely get a vaccine | 0.20094514 |
| Black | 45-54 | Male | High school or less | Probably get a vaccine | 0.06883197 |
| Black | 45-54 | Male | High school or less | Probably NOT get a vaccine | 0.07823318 |
| Black | 45-54 | Male | High school or less | Definitely NOT get a vaccine | 0.02786543 |
| Black | 45-54 | Male | High school or less | Already vaccinated | 0.15776481 |
| Black | 45-54 | Male | Incomplete college/Associate degree | Definitely get a vaccine | 0.10815254 |
| Black | 45-54 | Male | Incomplete college/Associate degree | Probably get a vaccine | 0.07793187 |
| Black | 45-54 | Male | Incomplete college/Associate degree | Probably NOT get a vaccine | 0.02135873 |
| Black | 45-54 | Male | Incomplete college/Associate degree | Definitely NOT get a vaccine | 0.01941395 |
| Black | 45-54 | Male | Incomplete college/Associate degree | Already vaccinated | 0.14445241 |
| Black | 45-54 | Male | Bachelor degree or higher | Definitely get a vaccine | 0.06964424 |
| Black | 45-54 | Male | Bachelor degree or higher | Probably get a vaccine | 0.04596581 |
| Black | 45-54 | Male | Bachelor degree or higher | Probably NOT get a vaccine | 0.00666862 |
| Black | 45-54 | Male | Bachelor degree or higher | Definitely NOT get a vaccine | 0.00118402 |
| Black | 45-54 | Male | Bachelor degree or higher | Already vaccinated | 0.12998950 |
| Black | 45-54 | Female | High school or less | Definitely get a vaccine | 0.14459036 |
| Black | 45-54 | Female | High school or less | Probably get a vaccine | 0.11687817 |
| Black | 45-54 | Female | High school or less | Probably NOT get a vaccine | 0.03644238 |
| Black | 45-54 | Female | High school or less | Definitely NOT get a vaccine | 0.03273686 |
| Black | 45-54 | Female | High school or less | Already vaccinated | 0.13599311 |
| Black | 45-54 | Female | Incomplete college/Associate degree | Definitely get a vaccine | 0.06573137 |
| Black | 45-54 | Female | Incomplete college/Associate degree | Probably get a vaccine | 0.08759151 |
| Black | 45-54 | Female | Incomplete college/Associate degree | Probably NOT get a vaccine | 0.04020254 |
| Black | 45-54 | Female | Incomplete college/Associate degree | Definitely NOT get a vaccine | 0.03154715 |
| Black | 45-54 | Female | Incomplete college/Associate degree | Already vaccinated | 0.17598599 |
| Black | 45-54 | Female | Bachelor degree or higher | Definitely get a vaccine | 0.04741024 |
| Black | 45-54 | Female | Bachelor degree or higher | Probably get a vaccine | 0.03366010 |
| Black | 45-54 | Female | Bachelor degree or higher | Probably NOT get a vaccine | 0.02546248 |
| Black | 45-54 | Female | Bachelor degree or higher | Definitely NOT get a vaccine | 0.00638455 |
| Black | 45-54 | Female | Bachelor degree or higher | Already vaccinated | 0.20653744 |
| Black | 55-64 | Male | High school or less | Definitely get a vaccine | 0.08532783 |
| Black | 55-64 | Male | High school or less | Probably get a vaccine | 0.11139014 |
| Black | 55-64 | Male | High school or less | Probably NOT get a vaccine | 0.05600410 |
| Black | 55-64 | Male | High school or less | Definitely NOT get a vaccine | 0.00908051 |
| Black | 55-64 | Male | High school or less | Already vaccinated | 0.22827449 |
| Black | 55-64 | Male | Incomplete college/Associate degree | Definitely get a vaccine | 0.07828084 |
| Black | 55-64 | Male | Incomplete college/Associate degree | Probably get a vaccine | 0.03651091 |
| Black | 55-64 | Male | Incomplete college/Associate degree | Probably NOT get a vaccine | 0.00437121 |
| Black | 55-64 | Male | Incomplete college/Associate degree | Definitely NOT get a vaccine | 0.00674703 |
| Black | 55-64 | Male | Incomplete college/Associate degree | Already vaccinated | 0.17262389 |
| Black | 55-64 | Male | Bachelor degree or higher | Definitely get a vaccine | 0.04867214 |
| Black | 55-64 | Male | Bachelor degree or higher | Probably get a vaccine | 0.01035086 |
| Black | 55-64 | Male | Bachelor degree or higher | Probably NOT get a vaccine | 0.00288946 |
| Black | 55-64 | Male | Bachelor degree or higher | Definitely NOT get a vaccine | 0.00398828 |
| Black | 55-64 | Male | Bachelor degree or higher | Already vaccinated | 0.13965130 |
| Black | 55-64 | Female | High school or less | Definitely get a vaccine | 0.12730308 |
| Black | 55-64 | Female | High school or less | Probably get a vaccine | 0.09843513 |
| Black | 55-64 | Female | High school or less | Probably NOT get a vaccine | 0.01891362 |
| Black | 55-64 | Female | High school or less | Definitely NOT get a vaccine | 0.01702204 |
| Black | 55-64 | Female | High school or less | Already vaccinated | 0.20194656 |
| Black | 55-64 | Female | Incomplete college/Associate degree | Definitely get a vaccine | 0.09857206 |
| Black | 55-64 | Female | Incomplete college/Associate degree | Probably get a vaccine | 0.06397370 |
| Black | 55-64 | Female | Incomplete college/Associate degree | Probably NOT get a vaccine | 0.02709497 |
| Black | 55-64 | Female | Incomplete college/Associate degree | Definitely NOT get a vaccine | 0.01041587 |
| Black | 55-64 | Female | Incomplete college/Associate degree | Already vaccinated | 0.21106210 |
| Black | 55-64 | Female | Bachelor degree or higher | Definitely get a vaccine | 0.05444974 |
| Black | 55-64 | Female | Bachelor degree or higher | Probably get a vaccine | 0.03155396 |
| Black | 55-64 | Female | Bachelor degree or higher | Probably NOT get a vaccine | 0.00737329 |
| Black | 55-64 | Female | Bachelor degree or higher | Definitely NOT get a vaccine | 0.00657467 |
| Black | 55-64 | Female | Bachelor degree or higher | Already vaccinated | 0.22033820 |
| Black | 65+ | Male | High school or less | Definitely get a vaccine | 0.02850046 |
| Black | 65+ | Male | High school or less | Probably get a vaccine | 0.02460403 |
| Black | 65+ | Male | High school or less | Probably NOT get a vaccine | 0.00210334 |
| Black | 65+ | Male | High school or less | Definitely NOT get a vaccine | 0.03370555 |
| Black | 65+ | Male | High school or less | Already vaccinated | 0.35494372 |
| Black | 65+ | Male | Incomplete college/Associate degree | Definitely get a vaccine | 0.02465111 |
| Black | 65+ | Male | Incomplete college/Associate degree | Probably get a vaccine | 0.01105626 |
| Black | 65+ | Male | Incomplete college/Associate degree | Probably NOT get a vaccine | 0.00153188 |
| Black | 65+ | Male | Incomplete college/Associate degree | Definitely NOT get a vaccine | 0.00303603 |
| Black | 65+ | Male | Incomplete college/Associate degree | Already vaccinated | 0.19647512 |
| Black | 65+ | Male | Bachelor degree or higher | Definitely get a vaccine | 0.01729118 |
| Black | 65+ | Male | Bachelor degree or higher | Probably get a vaccine | 0.00309439 |
| Black | 65+ | Male | Bachelor degree or higher | Probably NOT get a vaccine | 0.00049318 |
| Black | 65+ | Male | Bachelor degree or higher | Definitely NOT get a vaccine | 0.00067654 |
| Black | 65+ | Male | Bachelor degree or higher | Already vaccinated | 0.17602876 |
| Black | 65+ | Female | High school or less | Definitely get a vaccine | 0.12069507 |
| Black | 65+ | Female | High school or less | Probably get a vaccine | 0.01418060 |
| Black | 65+ | Female | High school or less | Probably NOT get a vaccine | 0.05566464 |
| Black | 65+ | Female | High school or less | Definitely NOT get a vaccine | 0.00510152 |
| Black | 65+ | Female | High school or less | Already vaccinated | 0.41330323 |
| Black | 65+ | Female | Incomplete college/Associate degree | Definitely get a vaccine | 0.03523853 |
| Black | 65+ | Female | Incomplete college/Associate degree | Probably get a vaccine | 0.01130142 |
| Black | 65+ | Female | Incomplete college/Associate degree | Probably NOT get a vaccine | 0.02107424 |
| Black | 65+ | Female | Incomplete college/Associate degree | Definitely NOT get a vaccine | 0.00313218 |
| Black | 65+ | Female | Incomplete college/Associate degree | Already vaccinated | 0.28064644 |
| Black | 65+ | Female | Bachelor degree or higher | Definitely get a vaccine | 0.01852849 |
| Black | 65+ | Female | Bachelor degree or higher | Probably get a vaccine | 0.00616889 |
| Black | 65+ | Female | Bachelor degree or higher | Probably NOT get a vaccine | 0.00309576 |
| Black | 65+ | Female | Bachelor degree or higher | Definitely NOT get a vaccine | 0.00262021 |
| Black | 65+ | Female | Bachelor degree or higher | Already vaccinated | 0.25469264 |
| Asian | 18-24 | Male | High school or less | Definitely get a vaccine | 0.08925343 |
| Asian | 18-24 | Male | High school or less | Probably get a vaccine | 0.05172164 |
| Asian | 18-24 | Male | High school or less | Probably NOT get a vaccine | 0.00514875 |
| Asian | 18-24 | Male | High school or less | Definitely NOT get a vaccine | 0.00957360 |
| Asian | 18-24 | Male | High school or less | Already vaccinated | 0.02536161 |
| Asian | 18-24 | Male | Incomplete college/Associate degree | Definitely get a vaccine | 0.05417718 |
| Asian | 18-24 | Male | Incomplete college/Associate degree | Probably get a vaccine | 0.01667725 |
| Asian | 18-24 | Male | Incomplete college/Associate degree | Probably NOT get a vaccine | 0.01107464 |
| Asian | 18-24 | Male | Incomplete college/Associate degree | Definitely NOT get a vaccine | 0.00000000 |
| Asian | 18-24 | Male | Incomplete college/Associate degree | Already vaccinated | 0.03983258 |
| Asian | 18-24 | Male | Bachelor degree or higher | Definitely get a vaccine | 0.03088524 |
| Asian | 18-24 | Male | Bachelor degree or higher | Probably get a vaccine | 0.00154429 |
| Asian | 18-24 | Male | Bachelor degree or higher | Probably NOT get a vaccine | 0.00077361 |
| Asian | 18-24 | Male | Bachelor degree or higher | Definitely NOT get a vaccine | 0.00000000 |
| Asian | 18-24 | Male | Bachelor degree or higher | Already vaccinated | 0.02231143 |
| Asian | 18-24 | Female | High school or less | Definitely get a vaccine | 0.03999595 |
| Asian | 18-24 | Female | High school or less | Probably get a vaccine | 0.01543754 |
| Asian | 18-24 | Female | High school or less | Probably NOT get a vaccine | 0.01152362 |
| Asian | 18-24 | Female | High school or less | Definitely NOT get a vaccine | 0.00000000 |
| Asian | 18-24 | Female | High school or less | Already vaccinated | 0.02519220 |
| Asian | 18-24 | Female | Incomplete college/Associate degree | Definitely get a vaccine | 0.07995502 |
| Asian | 18-24 | Female | Incomplete college/Associate degree | Probably get a vaccine | 0.01806746 |
| Asian | 18-24 | Female | Incomplete college/Associate degree | Probably NOT get a vaccine | 0.01214414 |
| Asian | 18-24 | Female | Incomplete college/Associate degree | Definitely NOT get a vaccine | 0.00000000 |
| Asian | 18-24 | Female | Incomplete college/Associate degree | Already vaccinated | 0.06719825 |
| Asian | 18-24 | Female | Bachelor degree or higher | Definitely get a vaccine | 0.01799504 |
| Asian | 18-24 | Female | Bachelor degree or higher | Probably get a vaccine | 0.01015665 |
| Asian | 18-24 | Female | Bachelor degree or higher | Probably NOT get a vaccine | 0.00155166 |
| Asian | 18-24 | Female | Bachelor degree or higher | Definitely NOT get a vaccine | 0.00000000 |
| Asian | 18-24 | Female | Bachelor degree or higher | Already vaccinated | 0.06306288 |
| Asian | 25-34 | Male | High school or less | Definitely get a vaccine | 0.02730611 |
| Asian | 25-34 | Male | High school or less | Probably get a vaccine | 0.02823729 |
| Asian | 25-34 | Male | High school or less | Probably NOT get a vaccine | 0.00000000 |
| Asian | 25-34 | Male | High school or less | Definitely NOT get a vaccine | 0.00387910 |
| Asian | 25-34 | Male | High school or less | Already vaccinated | 0.04056692 |
| Asian | 25-34 | Male | Incomplete college/Associate degree | Definitely get a vaccine | 0.03521256 |
| Asian | 25-34 | Male | Incomplete college/Associate degree | Probably get a vaccine | 0.04019436 |
| Asian | 25-34 | Male | Incomplete college/Associate degree | Probably NOT get a vaccine | 0.00265164 |
| Asian | 25-34 | Male | Incomplete college/Associate degree | Definitely NOT get a vaccine | 0.00197536 |
| Asian | 25-34 | Male | Incomplete college/Associate degree | Already vaccinated | 0.02133662 |
| Asian | 25-34 | Male | Bachelor degree or higher | Definitely get a vaccine | 0.14514023 |
| Asian | 25-34 | Male | Bachelor degree or higher | Probably get a vaccine | 0.03017174 |
| Asian | 25-34 | Male | Bachelor degree or higher | Probably NOT get a vaccine | 0.01254402 |
| Asian | 25-34 | Male | Bachelor degree or higher | Definitely NOT get a vaccine | 0.00033734 |
| Asian | 25-34 | Male | Bachelor degree or higher | Already vaccinated | 0.14795685 |
| Asian | 25-34 | Female | High school or less | Definitely get a vaccine | 0.06887820 |
| Asian | 25-34 | Female | High school or less | Probably get a vaccine | 0.01592830 |
| Asian | 25-34 | Female | High school or less | Probably NOT get a vaccine | 0.00752822 |
| Asian | 25-34 | Female | High school or less | Definitely NOT get a vaccine | 0.00401701 |
| Asian | 25-34 | Female | High school or less | Already vaccinated | 0.03726279 |
| Asian | 25-34 | Female | Incomplete college/Associate degree | Definitely get a vaccine | 0.02991411 |
| Asian | 25-34 | Female | Incomplete college/Associate degree | Probably get a vaccine | 0.01958547 |
| Asian | 25-34 | Female | Incomplete college/Associate degree | Probably NOT get a vaccine | 0.00092931 |
| Asian | 25-34 | Female | Incomplete college/Associate degree | Definitely NOT get a vaccine | 0.00000000 |
| Asian | 25-34 | Female | Incomplete college/Associate degree | Already vaccinated | 0.03250938 |
| Asian | 25-34 | Female | Bachelor degree or higher | Definitely get a vaccine | 0.15728934 |
| Asian | 25-34 | Female | Bachelor degree or higher | Probably get a vaccine | 0.02950131 |
| Asian | 25-34 | Female | Bachelor degree or higher | Probably NOT get a vaccine | 0.02915342 |
| Asian | 25-34 | Female | Bachelor degree or higher | Definitely NOT get a vaccine | 0.00514116 |
| Asian | 25-34 | Female | Bachelor degree or higher | Already vaccinated | 0.17112930 |
| Asian | 35-44 | Male | High school or less | Definitely get a vaccine | 0.06076729 |
| Asian | 35-44 | Male | High school or less | Probably get a vaccine | 0.05140866 |
| Asian | 35-44 | Male | High school or less | Probably NOT get a vaccine | 0.00277778 |
| Asian | 35-44 | Male | High school or less | Definitely NOT get a vaccine | 0.00000000 |
| Asian | 35-44 | Male | High school or less | Already vaccinated | 0.03048573 |
| Asian | 35-44 | Male | Incomplete college/Associate degree | Definitely get a vaccine | 0.01820107 |
| Asian | 35-44 | Male | Incomplete college/Associate degree | Probably get a vaccine | 0.01286178 |
| Asian | 35-44 | Male | Incomplete college/Associate degree | Probably NOT get a vaccine | 0.00547024 |
| Asian | 35-44 | Male | Incomplete college/Associate degree | Definitely NOT get a vaccine | 0.00133333 |
| Asian | 35-44 | Male | Incomplete college/Associate degree | Already vaccinated | 0.02919496 |
| Asian | 35-44 | Male | Bachelor degree or higher | Definitely get a vaccine | 0.14545323 |
| Asian | 35-44 | Male | Bachelor degree or higher | Probably get a vaccine | 0.02498438 |
| Asian | 35-44 | Male | Bachelor degree or higher | Probably NOT get a vaccine | 0.00712503 |
| Asian | 35-44 | Male | Bachelor degree or higher | Definitely NOT get a vaccine | 0.00180215 |
| Asian | 35-44 | Male | Bachelor degree or higher | Already vaccinated | 0.19059734 |
| Asian | 35-44 | Female | High school or less | Definitely get a vaccine | 0.04444953 |
| Asian | 35-44 | Female | High school or less | Probably get a vaccine | 0.03208350 |
| Asian | 35-44 | Female | High school or less | Probably NOT get a vaccine | 0.04197700 |
| Asian | 35-44 | Female | High school or less | Definitely NOT get a vaccine | 0.00213067 |
| Asian | 35-44 | Female | High school or less | Already vaccinated | 0.02019734 |
| Asian | 35-44 | Female | Incomplete college/Associate degree | Definitely get a vaccine | 0.02132005 |
| Asian | 35-44 | Female | Incomplete college/Associate degree | Probably get a vaccine | 0.01648900 |
| Asian | 35-44 | Female | Incomplete college/Associate degree | Probably NOT get a vaccine | 0.00595041 |
| Asian | 35-44 | Female | Incomplete college/Associate degree | Definitely NOT get a vaccine | 0.00315473 |
| Asian | 35-44 | Female | Incomplete college/Associate degree | Already vaccinated | 0.06090214 |
| Asian | 35-44 | Female | Bachelor degree or higher | Definitely get a vaccine | 0.10364696 |
| Asian | 35-44 | Female | Bachelor degree or higher | Probably get a vaccine | 0.03620420 |
| Asian | 35-44 | Female | Bachelor degree or higher | Probably NOT get a vaccine | 0.01210910 |
| Asian | 35-44 | Female | Bachelor degree or higher | Definitely NOT get a vaccine | 0.00608550 |
| Asian | 35-44 | Female | Bachelor degree or higher | Already vaccinated | 0.17777827 |
| Asian | 45-54 | Male | High school or less | Definitely get a vaccine | 0.10716453 |
| Asian | 45-54 | Male | High school or less | Probably get a vaccine | 0.00985087 |
| Asian | 45-54 | Male | High school or less | Probably NOT get a vaccine | 0.00000000 |
| Asian | 45-54 | Male | High school or less | Definitely NOT get a vaccine | 0.01794109 |
| Asian | 45-54 | Male | High school or less | Already vaccinated | 0.04811753 |
| Asian | 45-54 | Male | Incomplete college/Associate degree | Definitely get a vaccine | 0.05046298 |
| Asian | 45-54 | Male | Incomplete college/Associate degree | Probably get a vaccine | 0.00104424 |
| Asian | 45-54 | Male | Incomplete college/Associate degree | Probably NOT get a vaccine | 0.00221265 |
| Asian | 45-54 | Male | Incomplete college/Associate degree | Definitely NOT get a vaccine | 0.00000000 |
| Asian | 45-54 | Male | Incomplete college/Associate degree | Already vaccinated | 0.03858042 |
| Asian | 45-54 | Male | Bachelor degree or higher | Definitely get a vaccine | 0.08056107 |
| Asian | 45-54 | Male | Bachelor degree or higher | Probably get a vaccine | 0.02463886 |
| Asian | 45-54 | Male | Bachelor degree or higher | Probably NOT get a vaccine | 0.00107174 |
| Asian | 45-54 | Male | Bachelor degree or higher | Definitely NOT get a vaccine | 0.00255383 |
| Asian | 45-54 | Male | Bachelor degree or higher | Already vaccinated | 0.15283674 |
| Asian | 45-54 | Female | High school or less | Definitely get a vaccine | 0.06421435 |
| Asian | 45-54 | Female | High school or less | Probably get a vaccine | 0.00397683 |
| Asian | 45-54 | Female | High school or less | Probably NOT get a vaccine | 0.00082658 |
| Asian | 45-54 | Female | High school or less | Definitely NOT get a vaccine | 0.00513027 |
| Asian | 45-54 | Female | High school or less | Already vaccinated | 0.06932677 |
| Asian | 45-54 | Female | Incomplete college/Associate degree | Definitely get a vaccine | 0.05216686 |
| Asian | 45-54 | Female | Incomplete college/Associate degree | Probably get a vaccine | 0.00874338 |
| Asian | 45-54 | Female | Incomplete college/Associate degree | Probably NOT get a vaccine | 0.00089337 |
| Asian | 45-54 | Female | Incomplete college/Associate degree | Definitely NOT get a vaccine | 0.00158509 |
| Asian | 45-54 | Female | Incomplete college/Associate degree | Already vaccinated | 0.07866634 |
| Asian | 45-54 | Female | Bachelor degree or higher | Definitely get a vaccine | 0.06516548 |
| Asian | 45-54 | Female | Bachelor degree or higher | Probably get a vaccine | 0.01226359 |
| Asian | 45-54 | Female | Bachelor degree or higher | Probably NOT get a vaccine | 0.00606057 |
| Asian | 45-54 | Female | Bachelor degree or higher | Definitely NOT get a vaccine | 0.00276439 |
| Asian | 45-54 | Female | Bachelor degree or higher | Already vaccinated | 0.15696833 |
| Asian | 55-64 | Male | High school or less | Definitely get a vaccine | 0.06129467 |
| Asian | 55-64 | Male | High school or less | Probably get a vaccine | 0.02789364 |
| Asian | 55-64 | Male | High school or less | Probably NOT get a vaccine | 0.00000000 |
| Asian | 55-64 | Male | High school or less | Definitely NOT get a vaccine | 0.03074940 |
| Asian | 55-64 | Male | High school or less | Already vaccinated | 0.08722611 |
| Asian | 55-64 | Male | Incomplete college/Associate degree | Definitely get a vaccine | 0.03167597 |
| Asian | 55-64 | Male | Incomplete college/Associate degree | Probably get a vaccine | 0.00757007 |
| Asian | 55-64 | Male | Incomplete college/Associate degree | Probably NOT get a vaccine | 0.00231137 |
| Asian | 55-64 | Male | Incomplete college/Associate degree | Definitely NOT get a vaccine | 0.00083119 |
| Asian | 55-64 | Male | Incomplete college/Associate degree | Already vaccinated | 0.05368098 |
| Asian | 55-64 | Male | Bachelor degree or higher | Definitely get a vaccine | 0.06896146 |
| Asian | 55-64 | Male | Bachelor degree or higher | Probably get a vaccine | 0.00859401 |
| Asian | 55-64 | Male | Bachelor degree or higher | Probably NOT get a vaccine | 0.00280690 |
| Asian | 55-64 | Male | Bachelor degree or higher | Definitely NOT get a vaccine | 0.00360935 |
| Asian | 55-64 | Male | Bachelor degree or higher | Already vaccinated | 0.12885049 |
| Asian | 55-64 | Female | High school or less | Definitely get a vaccine | 0.03594074 |
| Asian | 55-64 | Female | High school or less | Probably get a vaccine | 0.00590746 |
| Asian | 55-64 | Female | High school or less | Probably NOT get a vaccine | 0.00122615 |
| Asian | 55-64 | Female | High school or less | Definitely NOT get a vaccine | 0.00283710 |
| Asian | 55-64 | Female | High school or less | Already vaccinated | 0.04762214 |
| Asian | 55-64 | Female | Incomplete college/Associate degree | Definitely get a vaccine | 0.04800200 |
| Asian | 55-64 | Female | Incomplete college/Associate degree | Probably get a vaccine | 0.00607689 |
| Asian | 55-64 | Female | Incomplete college/Associate degree | Probably NOT get a vaccine | 0.00182600 |
| Asian | 55-64 | Female | Incomplete college/Associate degree | Definitely NOT get a vaccine | 0.00077741 |
| Asian | 55-64 | Female | Incomplete college/Associate degree | Already vaccinated | 0.05289338 |
| Asian | 55-64 | Female | Bachelor degree or higher | Definitely get a vaccine | 0.07205372 |
| Asian | 55-64 | Female | Bachelor degree or higher | Probably get a vaccine | 0.00633430 |
| Asian | 55-64 | Female | Bachelor degree or higher | Probably NOT get a vaccine | 0.00148692 |
| Asian | 55-64 | Female | Bachelor degree or higher | Definitely NOT get a vaccine | 0.00420438 |
| Asian | 55-64 | Female | Bachelor degree or higher | Already vaccinated | 0.17571425 |
| Asian | 65+ | Male | High school or less | Definitely get a vaccine | 0.02843347 |
| Asian | 65+ | Male | High school or less | Probably get a vaccine | 0.00792447 |
| Asian | 65+ | Male | High school or less | Probably NOT get a vaccine | 0.00121173 |
| Asian | 65+ | Male | High school or less | Definitely NOT get a vaccine | 0.00562747 |
| Asian | 65+ | Male | High school or less | Already vaccinated | 0.11082763 |
| Asian | 65+ | Male | Incomplete college/Associate degree | Definitely get a vaccine | 0.00265349 |
| Asian | 65+ | Male | Incomplete college/Associate degree | Probably get a vaccine | 0.00005630 |
| Asian | 65+ | Male | Incomplete college/Associate degree | Probably NOT get a vaccine | 0.00000000 |
| Asian | 65+ | Male | Incomplete college/Associate degree | Definitely NOT get a vaccine | 0.00000000 |
| Asian | 65+ | Male | Incomplete college/Associate degree | Already vaccinated | 0.06814505 |
| Asian | 65+ | Male | Bachelor degree or higher | Definitely get a vaccine | 0.01674307 |
| Asian | 65+ | Male | Bachelor degree or higher | Probably get a vaccine | 0.00196322 |
| Asian | 65+ | Male | Bachelor degree or higher | Probably NOT get a vaccine | 0.00070353 |
| Asian | 65+ | Male | Bachelor degree or higher | Definitely NOT get a vaccine | 0.00729074 |
| Asian | 65+ | Male | Bachelor degree or higher | Already vaccinated | 0.17292570 |
| Asian | 65+ | Female | High school or less | Definitely get a vaccine | 0.04878502 |
| Asian | 65+ | Female | High school or less | Probably get a vaccine | 0.00072172 |
| Asian | 65+ | Female | High school or less | Probably NOT get a vaccine | 0.00048115 |
| Asian | 65+ | Female | High school or less | Definitely NOT get a vaccine | 0.00176244 |
| Asian | 65+ | Female | High school or less | Already vaccinated | 0.15986237 |
| Asian | 65+ | Female | Incomplete college/Associate degree | Definitely get a vaccine | 0.01266344 |
| Asian | 65+ | Female | Incomplete college/Associate degree | Probably get a vaccine | 0.00101169 |
| Asian | 65+ | Female | Incomplete college/Associate degree | Probably NOT get a vaccine | 0.00075256 |
| Asian | 65+ | Female | Incomplete college/Associate degree | Definitely NOT get a vaccine | 0.00115714 |
| Asian | 65+ | Female | Incomplete college/Associate degree | Already vaccinated | 0.07141849 |
| Asian | 65+ | Female | Bachelor degree or higher | Definitely get a vaccine | 0.00530137 |
| Asian | 65+ | Female | Bachelor degree or higher | Probably get a vaccine | 0.00299273 |
| Asian | 65+ | Female | Bachelor degree or higher | Probably NOT get a vaccine | 0.00551290 |
| Asian | 65+ | Female | Bachelor degree or higher | Definitely NOT get a vaccine | 0.00162664 |
| Asian | 65+ | Female | Bachelor degree or higher | Already vaccinated | 0.14017583 |
| Other | 18-24 | Male | High school or less | Definitely get a vaccine | 0.02134556 |
| Other | 18-24 | Male | High school or less | Probably get a vaccine | 0.03484817 |
| Other | 18-24 | Male | High school or less | Probably NOT get a vaccine | 0.03695260 |
| Other | 18-24 | Male | High school or less | Definitely NOT get a vaccine | 0.01833893 |
| Other | 18-24 | Male | High school or less | Already vaccinated | 0.01652596 |
| Other | 18-24 | Male | Incomplete college/Associate degree | Definitely get a vaccine | 0.13181543 |
| Other | 18-24 | Male | Incomplete college/Associate degree | Probably get a vaccine | 0.00927891 |
| Other | 18-24 | Male | Incomplete college/Associate degree | Probably NOT get a vaccine | 0.00472744 |
| Other | 18-24 | Male | Incomplete college/Associate degree | Definitely NOT get a vaccine | 0.01585518 |
| Other | 18-24 | Male | Incomplete college/Associate degree | Already vaccinated | 0.00712855 |
| Other | 18-24 | Male | Bachelor degree or higher | Definitely get a vaccine | 0.02276208 |
| Other | 18-24 | Male | Bachelor degree or higher | Probably get a vaccine | 0.00385456 |
| Other | 18-24 | Male | Bachelor degree or higher | Probably NOT get a vaccine | 0.00000000 |
| Other | 18-24 | Male | Bachelor degree or higher | Definitely NOT get a vaccine | 0.00000000 |
| Other | 18-24 | Male | Bachelor degree or higher | Already vaccinated | 0.00500574 |
| Other | 18-24 | Female | High school or less | Definitely get a vaccine | 0.09231015 |
| Other | 18-24 | Female | High school or less | Probably get a vaccine | 0.03309172 |
| Other | 18-24 | Female | High school or less | Probably NOT get a vaccine | 0.01209313 |
| Other | 18-24 | Female | High school or less | Definitely NOT get a vaccine | 0.02633066 |
| Other | 18-24 | Female | High school or less | Already vaccinated | 0.00833759 |
| Other | 18-24 | Female | Incomplete college/Associate degree | Definitely get a vaccine | 0.07724159 |
| Other | 18-24 | Female | Incomplete college/Associate degree | Probably get a vaccine | 0.02892457 |
| Other | 18-24 | Female | Incomplete college/Associate degree | Probably NOT get a vaccine | 0.02408120 |
| Other | 18-24 | Female | Incomplete college/Associate degree | Definitely NOT get a vaccine | 0.00716477 |
| Other | 18-24 | Female | Incomplete college/Associate degree | Already vaccinated | 0.04024077 |
| Other | 18-24 | Female | Bachelor degree or higher | Definitely get a vaccine | 0.00539392 |
| Other | 18-24 | Female | Bachelor degree or higher | Probably get a vaccine | 0.00008376 |
| Other | 18-24 | Female | Bachelor degree or higher | Probably NOT get a vaccine | 0.00000000 |
| Other | 18-24 | Female | Bachelor degree or higher | Definitely NOT get a vaccine | 0.00088037 |
| Other | 18-24 | Female | Bachelor degree or higher | Already vaccinated | 0.01093880 |
| Other | 25-34 | Male | High school or less | Definitely get a vaccine | 0.12776446 |
| Other | 25-34 | Male | High school or less | Probably get a vaccine | 0.07900280 |
| Other | 25-34 | Male | High school or less | Probably NOT get a vaccine | 0.06186034 |
| Other | 25-34 | Male | High school or less | Definitely NOT get a vaccine | 0.01137841 |
| Other | 25-34 | Male | High school or less | Already vaccinated | 0.03519610 |
| Other | 25-34 | Male | Incomplete college/Associate degree | Definitely get a vaccine | 0.05805614 |
| Other | 25-34 | Male | Incomplete college/Associate degree | Probably get a vaccine | 0.01644711 |
| Other | 25-34 | Male | Incomplete college/Associate degree | Probably NOT get a vaccine | 0.01484882 |
| Other | 25-34 | Male | Incomplete college/Associate degree | Definitely NOT get a vaccine | 0.01232191 |
| Other | 25-34 | Male | Incomplete college/Associate degree | Already vaccinated | 0.02560084 |
| Other | 25-34 | Male | Bachelor degree or higher | Definitely get a vaccine | 0.05096770 |
| Other | 25-34 | Male | Bachelor degree or higher | Probably get a vaccine | 0.02045735 |
| Other | 25-34 | Male | Bachelor degree or higher | Probably NOT get a vaccine | 0.00743444 |
| Other | 25-34 | Male | Bachelor degree or higher | Definitely NOT get a vaccine | 0.01098614 |
| Other | 25-34 | Male | Bachelor degree or higher | Already vaccinated | 0.05757666 |
| Other | 25-34 | Female | High school or less | Definitely get a vaccine | 0.04382529 |
| Other | 25-34 | Female | High school or less | Probably get a vaccine | 0.06850471 |
| Other | 25-34 | Female | High school or less | Probably NOT get a vaccine | 0.03456258 |
| Other | 25-34 | Female | High school or less | Definitely NOT get a vaccine | 0.02769851 |
| Other | 25-34 | Female | High school or less | Already vaccinated | 0.01326296 |
| Other | 25-34 | Female | Incomplete college/Associate degree | Definitely get a vaccine | 0.07752993 |
| Other | 25-34 | Female | Incomplete college/Associate degree | Probably get a vaccine | 0.03673761 |
| Other | 25-34 | Female | Incomplete college/Associate degree | Probably NOT get a vaccine | 0.03803600 |
| Other | 25-34 | Female | Incomplete college/Associate degree | Definitely NOT get a vaccine | 0.02936455 |
| Other | 25-34 | Female | Incomplete college/Associate degree | Already vaccinated | 0.05876022 |
| Other | 25-34 | Female | Bachelor degree or higher | Definitely get a vaccine | 0.04771763 |
| Other | 25-34 | Female | Bachelor degree or higher | Probably get a vaccine | 0.01873126 |
| Other | 25-34 | Female | Bachelor degree or higher | Probably NOT get a vaccine | 0.03577077 |
| Other | 25-34 | Female | Bachelor degree or higher | Definitely NOT get a vaccine | 0.00675523 |
| Other | 25-34 | Female | Bachelor degree or higher | Already vaccinated | 0.10785677 |
| Other | 35-44 | Male | High school or less | Definitely get a vaccine | 0.08474768 |
| Other | 35-44 | Male | High school or less | Probably get a vaccine | 0.04134865 |
| Other | 35-44 | Male | High school or less | Probably NOT get a vaccine | 0.05452959 |
| Other | 35-44 | Male | High school or less | Definitely NOT get a vaccine | 0.04386125 |
| Other | 35-44 | Male | High school or less | Already vaccinated | 0.05940614 |
| Other | 35-44 | Male | Incomplete college/Associate degree | Definitely get a vaccine | 0.04576201 |
| Other | 35-44 | Male | Incomplete college/Associate degree | Probably get a vaccine | 0.02808035 |
| Other | 35-44 | Male | Incomplete college/Associate degree | Probably NOT get a vaccine | 0.00842230 |
| Other | 35-44 | Male | Incomplete college/Associate degree | Definitely NOT get a vaccine | 0.02271898 |
| Other | 35-44 | Male | Incomplete college/Associate degree | Already vaccinated | 0.07699941 |
| Other | 35-44 | Male | Bachelor degree or higher | Definitely get a vaccine | 0.02270526 |
| Other | 35-44 | Male | Bachelor degree or higher | Probably get a vaccine | 0.00743178 |
| Other | 35-44 | Male | Bachelor degree or higher | Probably NOT get a vaccine | 0.02267151 |
| Other | 35-44 | Male | Bachelor degree or higher | Definitely NOT get a vaccine | 0.00992088 |
| Other | 35-44 | Male | Bachelor degree or higher | Already vaccinated | 0.06387236 |
| Other | 35-44 | Female | High school or less | Definitely get a vaccine | 0.04523140 |
| Other | 35-44 | Female | High school or less | Probably get a vaccine | 0.07287856 |
| Other | 35-44 | Female | High school or less | Probably NOT get a vaccine | 0.02514067 |
| Other | 35-44 | Female | High school or less | Definitely NOT get a vaccine | 0.05906947 |
| Other | 35-44 | Female | High school or less | Already vaccinated | 0.06503431 |
| Other | 35-44 | Female | Incomplete college/Associate degree | Definitely get a vaccine | 0.04828341 |
| Other | 35-44 | Female | Incomplete college/Associate degree | Probably get a vaccine | 0.02150047 |
| Other | 35-44 | Female | Incomplete college/Associate degree | Probably NOT get a vaccine | 0.03593946 |
| Other | 35-44 | Female | Incomplete college/Associate degree | Definitely NOT get a vaccine | 0.03758369 |
| Other | 35-44 | Female | Incomplete college/Associate degree | Already vaccinated | 0.07525337 |
| Other | 35-44 | Female | Bachelor degree or higher | Definitely get a vaccine | 0.03592234 |
| Other | 35-44 | Female | Bachelor degree or higher | Probably get a vaccine | 0.01493844 |
| Other | 35-44 | Female | Bachelor degree or higher | Probably NOT get a vaccine | 0.01784177 |
| Other | 35-44 | Female | Bachelor degree or higher | Definitely NOT get a vaccine | 0.01219948 |
| Other | 35-44 | Female | Bachelor degree or higher | Already vaccinated | 0.07802971 |
| Other | 45-54 | Male | High school or less | Definitely get a vaccine | 0.08802198 |
| Other | 45-54 | Male | High school or less | Probably get a vaccine | 0.03502189 |
| Other | 45-54 | Male | High school or less | Probably NOT get a vaccine | 0.07402769 |
| Other | 45-54 | Male | High school or less | Definitely NOT get a vaccine | 0.05621023 |
| Other | 45-54 | Male | High school or less | Already vaccinated | 0.13367274 |
| Other | 45-54 | Male | Incomplete college/Associate degree | Definitely get a vaccine | 0.00839404 |
| Other | 45-54 | Male | Incomplete college/Associate degree | Probably get a vaccine | 0.02700370 |
| Other | 45-54 | Male | Incomplete college/Associate degree | Probably NOT get a vaccine | 0.00527595 |
| Other | 45-54 | Male | Incomplete college/Associate degree | Definitely NOT get a vaccine | 0.01321674 |
| Other | 45-54 | Male | Incomplete college/Associate degree | Already vaccinated | 0.05811513 |
| Other | 45-54 | Male | Bachelor degree or higher | Definitely get a vaccine | 0.02790177 |
| Other | 45-54 | Male | Bachelor degree or higher | Probably get a vaccine | 0.00501501 |
| Other | 45-54 | Male | Bachelor degree or higher | Probably NOT get a vaccine | 0.01020867 |
| Other | 45-54 | Male | Bachelor degree or higher | Definitely NOT get a vaccine | 0.01779168 |
| Other | 45-54 | Male | Bachelor degree or higher | Already vaccinated | 0.04079571 |
| Other | 45-54 | Female | High school or less | Definitely get a vaccine | 0.07911984 |
| Other | 45-54 | Female | High school or less | Probably get a vaccine | 0.03025230 |
| Other | 45-54 | Female | High school or less | Probably NOT get a vaccine | 0.02096736 |
| Other | 45-54 | Female | High school or less | Definitely NOT get a vaccine | 0.01936923 |
| Other | 45-54 | Female | High school or less | Already vaccinated | 0.08342101 |
| Other | 45-54 | Female | Incomplete college/Associate degree | Definitely get a vaccine | 0.02646384 |
| Other | 45-54 | Female | Incomplete college/Associate degree | Probably get a vaccine | 0.01233945 |
| Other | 45-54 | Female | Incomplete college/Associate degree | Probably NOT get a vaccine | 0.01221315 |
| Other | 45-54 | Female | Incomplete college/Associate degree | Definitely NOT get a vaccine | 0.02329923 |
| Other | 45-54 | Female | Incomplete college/Associate degree | Already vaccinated | 0.05832196 |
| Other | 45-54 | Female | Bachelor degree or higher | Definitely get a vaccine | 0.01531184 |
| Other | 45-54 | Female | Bachelor degree or higher | Probably get a vaccine | 0.00782408 |
| Other | 45-54 | Female | Bachelor degree or higher | Probably NOT get a vaccine | 0.00359570 |
| Other | 45-54 | Female | Bachelor degree or higher | Definitely NOT get a vaccine | 0.00559669 |
| Other | 45-54 | Female | Bachelor degree or higher | Already vaccinated | 0.06362495 |
| Other | 55-64 | Male | High school or less | Definitely get a vaccine | 0.07095152 |
| Other | 55-64 | Male | High school or less | Probably get a vaccine | 0.01693015 |
| Other | 55-64 | Male | High school or less | Probably NOT get a vaccine | 0.04932473 |
| Other | 55-64 | Male | High school or less | Definitely NOT get a vaccine | 0.01437207 |
| Other | 55-64 | Male | High school or less | Already vaccinated | 0.06810377 |
| Other | 55-64 | Male | Incomplete college/Associate degree | Definitely get a vaccine | 0.02758296 |
| Other | 55-64 | Male | Incomplete college/Associate degree | Probably get a vaccine | 0.01715118 |
| Other | 55-64 | Male | Incomplete college/Associate degree | Probably NOT get a vaccine | 0.02807585 |
| Other | 55-64 | Male | Incomplete college/Associate degree | Definitely NOT get a vaccine | 0.00918048 |
| Other | 55-64 | Male | Incomplete college/Associate degree | Already vaccinated | 0.04021316 |
| Other | 55-64 | Male | Bachelor degree or higher | Definitely get a vaccine | 0.01018427 |
| Other | 55-64 | Male | Bachelor degree or higher | Probably get a vaccine | 0.00348903 |
| Other | 55-64 | Male | Bachelor degree or higher | Probably NOT get a vaccine | 0.01075005 |
| Other | 55-64 | Male | Bachelor degree or higher | Definitely NOT get a vaccine | 0.00549751 |
| Other | 55-64 | Male | Bachelor degree or higher | Already vaccinated | 0.02429029 |
| Other | 55-64 | Female | High school or less | Definitely get a vaccine | 0.03847726 |
| Other | 55-64 | Female | High school or less | Probably get a vaccine | 0.03885213 |
| Other | 55-64 | Female | High school or less | Probably NOT get a vaccine | 0.00281373 |
| Other | 55-64 | Female | High school or less | Definitely NOT get a vaccine | 0.01573796 |
| Other | 55-64 | Female | High school or less | Already vaccinated | 0.07249836 |
| Other | 55-64 | Female | Incomplete college/Associate degree | Definitely get a vaccine | 0.04025226 |
| Other | 55-64 | Female | Incomplete college/Associate degree | Probably get a vaccine | 0.02057520 |
| Other | 55-64 | Female | Incomplete college/Associate degree | Probably NOT get a vaccine | 0.01498319 |
| Other | 55-64 | Female | Incomplete college/Associate degree | Definitely NOT get a vaccine | 0.01011756 |
| Other | 55-64 | Female | Incomplete college/Associate degree | Already vaccinated | 0.04652537 |
| Other | 55-64 | Female | Bachelor degree or higher | Definitely get a vaccine | 0.01765683 |
| Other | 55-64 | Female | Bachelor degree or higher | Probably get a vaccine | 0.00622235 |
| Other | 55-64 | Female | Bachelor degree or higher | Probably NOT get a vaccine | 0.00186721 |
| Other | 55-64 | Female | Bachelor degree or higher | Definitely NOT get a vaccine | 0.01312964 |
| Other | 55-64 | Female | Bachelor degree or higher | Already vaccinated | 0.06076447 |
| Other | 65+ | Male | High school or less | Definitely get a vaccine | 0.03506804 |
| Other | 65+ | Male | High school or less | Probably get a vaccine | 0.00710667 |
| Other | 65+ | Male | High school or less | Probably NOT get a vaccine | 0.00337353 |
| Other | 65+ | Male | High school or less | Definitely NOT get a vaccine | 0.04983411 |
| Other | 65+ | Male | High school or less | Already vaccinated | 0.11919767 |
| Other | 65+ | Male | Incomplete college/Associate degree | Definitely get a vaccine | 0.00624257 |
| Other | 65+ | Male | Incomplete college/Associate degree | Probably get a vaccine | 0.00445378 |
| Other | 65+ | Male | Incomplete college/Associate degree | Probably NOT get a vaccine | 0.00341884 |
| Other | 65+ | Male | Incomplete college/Associate degree | Definitely NOT get a vaccine | 0.00261004 |
| Other | 65+ | Male | Incomplete college/Associate degree | Already vaccinated | 0.05964538 |
| Other | 65+ | Male | Bachelor degree or higher | Definitely get a vaccine | 0.00243740 |
| Other | 65+ | Male | Bachelor degree or higher | Probably get a vaccine | 0.00479646 |
| Other | 65+ | Male | Bachelor degree or higher | Probably NOT get a vaccine | 0.00089168 |
| Other | 65+ | Male | Bachelor degree or higher | Definitely NOT get a vaccine | 0.01960215 |
| Other | 65+ | Male | Bachelor degree or higher | Already vaccinated | 0.06006961 |
| Other | 65+ | Female | High school or less | Definitely get a vaccine | 0.03970670 |
| Other | 65+ | Female | High school or less | Probably get a vaccine | 0.01987238 |
| Other | 65+ | Female | High school or less | Probably NOT get a vaccine | 0.00858451 |
| Other | 65+ | Female | High school or less | Definitely NOT get a vaccine | 0.01021777 |
| Other | 65+ | Female | High school or less | Already vaccinated | 0.12835556 |
| Other | 65+ | Female | Incomplete college/Associate degree | Definitely get a vaccine | 0.02009652 |
| Other | 65+ | Female | Incomplete college/Associate degree | Probably get a vaccine | 0.00649460 |
| Other | 65+ | Female | Incomplete college/Associate degree | Probably NOT get a vaccine | 0.00437677 |
| Other | 65+ | Female | Incomplete college/Associate degree | Definitely NOT get a vaccine | 0.00429728 |
| Other | 65+ | Female | Incomplete college/Associate degree | Already vaccinated | 0.07079219 |
| Other | 65+ | Female | Bachelor degree or higher | Definitely get a vaccine | 0.00763137 |
| Other | 65+ | Female | Bachelor degree or higher | Probably get a vaccine | 0.00315138 |
| Other | 65+ | Female | Bachelor degree or higher | Probably NOT get a vaccine | 0.00484022 |
| Other | 65+ | Female | Bachelor degree or higher | Definitely NOT get a vaccine | 0.00645835 |
| Other | 65+ | Female | Bachelor degree or higher | Already vaccinated | 0.06108241 |
