# Supplementary material for: Preferences for COVID-19 vaccine distribution strategies in the US: A discrete choice survey
Source: PLoS One. 2021 Aug 20;16(8):e0256394. doi: 10.1371/journal.pone.0256394 (PMC8378751; doi:10.1371/journal.pone.0256394)
Supplement: S2 Table — (DOCX) [file pone.0256394.s002.docx]

**S2 Table: Weighted mean preferences – total population**

| Attribute | Relative utilities | | | | Standard deviation | | | |  |
| --- | --- | --- | --- | --- | --- | --- | --- | --- | --- |
|  | Utility | Low CI | High CI | p-value | SD | Low CI | High CI | p-value | |
| Opt-out | -1.77 | -1.96 | -1.58 | <0.001 | 0.50 | 0.21 | 0.79 | 0.001 | |
| Vaccinate at pharmacy vs. health center | 0.07 | -0.04 | 0.19 | 0.222 | 0.65 | 0.39 | 0.90 | <0.001 | |
| Vaccinate at community venue vs. health center | -0.13 | -0.24 | -0.02 | 0.022 | 0.93 | 0.74 | 1.13 | <0.001 | |
| Vaccinate at home vs. health center | -0.10 | -0.27 | 0.07 | 0.239 | 0.69 | 0.51 | 0.87 | <0.001 | |
| Vaccinate at mass site vs. health center | -0.29 | -0.39 | -0.18 | <0.001 | -0.24 | -0.64 | 0.15 | 0.221 | |
| Wait for 1 hr vs. immediate service | -0.21 | -0.32 | -0.11 | <0.001 | 0.53 | 0.35 | 0.71 | <0.001 | |
| Wait for 2 hrs vs. immediate service | -0.54 | -0.64 | -0.43 | <0.001 | 0.53 | 0.37 | 0.70 | <0.001 | |
| Phone vs. online appointment booking | -0.06 | -0.15 | 0.02 | 0.145 | 0.41 | 0.18 | 0.63 | <0.001 | |
| Drop in (no booking) vs. online appointment booking | 0.05 | -0.03 | 0.13 | 0.251 | 1.07 | 0.97 | 1.18 | <0.001 | |
| Vaccinate annually vs. once | -0.79 | -0.89 | -0.70 | <0.001 | 0.23 | -0.13 | 0.60 | 0.209 | |
| Enforcement for air travel vs. no enforcement | -0.13 | -0.23 | -0.04 | 0.006 | -0.40 | -0.64 | -0.17 | 0.001 | |
| Enforcement for work/school vs. no enforcement | -0.20 | -0.30 | -0.10 | <0.001 | -0.22 | -0.50 | 0.06 | 0.126 | |
| Enforcement for recreation vs. no enforcement | -0.05 | -0.15 | 0.05 | 0.298 | 0.36 | 0.06 | 0.65 | 0.017 | |
| A few in the community vaccinated vs. no one | 0.36 | 0.28 | 0.43 | <0.001 | -0.49 | -0.66 | -0.32 | <0.001 | |
| Almost everyone in the community vaccinated vs. no one | 0.48 | 0.39 | 0.56 | <0.001 | 0.97 | 0.83 | 1.10 | <0.001 | |
| Two vaccine doses vs. a single dose | -0.29 | -0.37 | -0.20 | <0.001 | 0.50 | 0.21 | 0.79 | 0.001 | |
