# Supplementary material for: Preferences for COVID-19 vaccine distribution strategies in the US: A discrete choice survey
Source: PLoS One. 2021 Aug 20;16(8):e0256394. doi: 10.1371/journal.pone.0256394 (PMC8378751; doi:10.1371/journal.pone.0256394)
Supplement: S3 Table — (DOCX) [file pone.0256394.s003.docx]

S3 Table(a): Weighted mean preferences – Already vaccinated

| Attribute | Relative utilities | | | | Standard deviation | | | |
| --- | --- | --- | --- | --- | --- | --- | --- | --- |
|  | Utility | Low CI | High CI | p-value | SD | Low CI | High CI | p-value |
| Opt-out | -1.79 | -2.10 | -1.49 | <0.001 |  |  |  |  |
| Vaccinate at pharmacy vs. health center | 0.17 | -0.03 | 0.37 | 0.089 | -0.30 | -1.02 | 0.43 | 0.422 |
| Vaccinate at community venue vs. health center | -0.09 | -0.27 | 0.08 | 0.300 | 0.72 | 0.36 | 1.07 | <0.001 |
| Vaccinate at home vs. health center | -0.01 | -0.29 | 0.27 | 0.939 | 1.08 | 0.78 | 1.37 | <0.001 |
| Vaccinate at mass site vs. health center | -0.31 | -0.48 | -0.14 | <0.001 | 0.69 | 0.41 | 0.97 | <0.001 |
| Wait for 1 hr vs. immediate service | -0.20 | -0.37 | -0.02 | 0.027 | 0.39 | 0.07 | 0.72 | 0.017 |
| Wait for 2 hrs vs. immediate service | -0.65 | -0.84 | -0.47 | <0.001 | 0.82 | 0.44 | 1.20 | <0.001 |
| Phone vs. online appointment booking | -0.09 | -0.23 | 0.05 | 0.201 | 0.56 | 0.28 | 0.84 | <0.001 |
| Drop in (no booking) vs. online appointment booking | 0.14 | 0.00 | 0.27 | 0.044 | 0.46 | 0.13 | 0.79 | 0.007 |
| Vaccinate annually vs. once | -0.96 | -1.13 | -0.80 | <0.001 | 1.10 | 0.95 | 1.25 | <0.001 |
| Enforcement for air travel vs. no enforcement | 0.01 | -0.14 | 0.17 | 0.879 | -0.27 | -0.89 | 0.35 | 0.399 |
| Enforcement for work/school vs. no enforcement | -0.14 | -0.31 | 0.03 | 0.110 | -0.38 | -0.69 | -0.07 | 0.015 |
| Enforcement for recreation vs. no enforcement | 0.10 | -0.05 | 0.25 | 0.211 | -0.07 | -0.56 | 0.41 | 0.769 |
| A few in the community vaccinated vs. no one | 0.45 | 0.34 | 0.57 | <0.001 | 0.16 | -0.06 | 0.37 | 0.152 |
| Almost everyone in the community vaccinated vs. no one | 0.57 | 0.44 | 0.70 | <0.001 | -0.42 | -0.75 | -0.08 | 0.015 |
| Two vaccine doses vs. a single dose | -0.13 | -0.30 | 0.03 | 0.112 | 1.10 | 0.83 | 1.38 | <0.001 |

S3 Table (b): Weighted mean preferences – Definitely get vaccinated

| Attribute | Relative utilities | | | | Standard deviation | | | |
| --- | --- | --- | --- | --- | --- | --- | --- | --- |
|  | Utility | Low CI | High CI | p-value | SD | Low CI | High CI | p-value |
| Opt-out | -2.34 | -2.67 | -2.02 | <0.001 |  |  |  |  |
| Vaccinate at pharmacy vs. health center | -0.01 | -0.21 | 0.20 | 0.935 | 0.73 | 0.32 | 1.14 | <0.001 |
| Vaccinate at community venue vs. health center | -0.08 | -0.27 | 0.11 | 0.402 | 0.60 | 0.17 | 1.03 | 0.006 |
| Vaccinate at home vs. health center | -0.05 | -0.32 | 0.22 | 0.728 | 0.84 | 0.48 | 1.20 | <0.001 |
| Vaccinate at mass site vs. health center | -0.49 | -0.71 | -0.27 | <0.001 | 0.92 | 0.69 | 1.16 | <0.001 |
| Wait for 1 hr vs. immediate service | -0.28 | -0.46 | -0.10 | 0.003 | -0.03 | -0.51 | 0.46 | 0.912 |
| Wait for 2 hrs vs. immediate service | -0.58 | -0.78 | -0.37 | <0.001 | 0.49 | 0.15 | 0.83 | 0.004 |
| Phone vs. online appointment booking | 0.01 | -0.14 | 0.15 | 0.924 | -0.36 | -0.70 | -0.02 | 0.037 |
| Drop in (no booking) vs. online appointment booking | 0.07 | -0.09 | 0.23 | 0.370 | 0.54 | 0.22 | 0.86 | 0.001 |
| Vaccinate annually vs. once | -0.69 | -0.85 | -0.53 | <0.001 | 1.05 | 0.88 | 1.23 | <0.001 |
| Enforcement for air travel vs. no enforcement | -0.04 | -0.20 | 0.13 | 0.654 | 0.18 | -1.30 | 1.66 | 0.813 |
| Enforcement for work/school vs. no enforcement | -0.04 | -0.22 | 0.14 | 0.671 | 0.48 | -0.35 | 1.31 | 0.256 |
| Enforcement for recreation vs. no enforcement | 0.11 | -0.08 | 0.29 | 0.251 | 0.07 | -0.47 | 0.62 | 0.787 |
| A few in the community vaccinated vs. no one | 0.43 | 0.28 | 0.59 | <0.001 | 0.31 | -0.25 | 0.88 | 0.280 |
| Almost everyone in the community vaccinated vs. no one | 0.50 | 0.33 | 0.68 | <0.001 | 0.57 | 0.17 | 0.98 | 0.006 |
| Two vaccine doses vs. a single dose | -0.41 | -0.55 | -0.27 | <0.001 | 0.76 | 0.51 | 1.01 | <0.001 |

S3 Table (c): Weighted mean preferences – Probably get vaccinated

| Attribute | Relative utilities | | | | Standard deviation | | | |  |
| --- | --- | --- | --- | --- | --- | --- | --- | --- | --- |
|  | Utility | Low CI | High CI | p-value | SD | Low CI | High CI | p-value | |
| Opt-out | -1.79 | -2.29 | -1.29 | <0.001 |  |  |  |  | |
| Vaccinate at pharmacy vs. health center | 0.06 | -0.18 | 0.30 | 0.614 | -0.61 | -0.96 | -0.27 | 0.001 | |
| Vaccinate at community venue vs. health center | -0.18 | -0.45 | 0.09 | 0.201 | 0.93 | 0.57 | 1.30 | <0.001 | |
| Vaccinate at home vs. health center | -0.13 | -0.56 | 0.30 | 0.557 | 1.15 | 0.63 | 1.66 | <0.001 | |
| Vaccinate at mass site vs. health center | -0.20 | -0.45 | 0.06 | 0.133 | 0.66 | 0.06 | 1.25 | 0.030 | |
| Wait for 1 hr vs. immediate service | -0.17 | -0.42 | 0.08 | 0.185 | -0.37 | -0.63 | -0.11 | 0.005 | |
| Wait for 2 hrs vs. immediate service | -0.23 | -0.49 | 0.03 | 0.078 | -0.41 | -0.80 | -0.02 | 0.041 | |
| Phone vs. online appointment booking | -0.11 | -0.34 | 0.12 | 0.361 | -0.52 | -0.79 | -0.25 | <0.001 | |
| Drop in (no booking) vs. online appointment booking | -0.06 | -0.27 | 0.15 | 0.587 | -0.17 | -0.48 | 0.15 | 0.300 | |
| Vaccinate annually vs. once | -0.58 | -0.81 | -0.36 | <0.001 | 1.10 | 0.83 | 1.37 | <0.001 | |
| Enforcement for air travel vs. no enforcement | -0.43 | -0.68 | -0.19 | <0.001 | -0.65 | -1.03 | -0.28 | 0.001 | |
| Enforcement for work/school vs. no enforcement | -0.33 | -0.56 | -0.09 | 0.007 | 0.78 | 0.36 | 1.21 | <0.001 | |
| Enforcement for recreation vs. no enforcement | -0.41 | -0.67 | -0.16 | 0.002 | 0.25 | -0.28 | 0.78 | 0.361 | |
| A few in the community vaccinated vs. no one | 0.25 | 0.07 | 0.43 | 0.006 | 0.27 | -0.27 | 0.80 | 0.324 | |
| Almost everyone in the community vaccinated vs. no one | 0.39 | 0.18 | 0.60 | <0.001 | -0.10 | -0.67 | 0.47 | 0.729 | |
| Two vaccine doses vs. a single dose | -0.43 | -0.63 | -0.22 | <0.001 | 1.12 | 0.87 | 1.37 | <0.001 | |

S3 Table (d): Weighted mean preferences – Probably NOT get vaccinated

| Attribute | Relative utilities | | | | Standard deviation | | | |  |
| --- | --- | --- | --- | --- | --- | --- | --- | --- | --- |
|  | Utility | Low CI | High CI | p-value | SD | Low CI | High CI | p-value | |
| Opt-out | -1.68 | -2.20 | -1.15 | <0.001 |  |  |  |  | |
| Vaccinate at pharmacy vs. health center | -0.09 | -0.42 | 0.24 | 0.580 | 0.12 | -0.41 | 0.66 | 0.655 | |
| Vaccinate at community venue vs. health center | -0.40 | -0.87 | 0.07 | 0.096 | 1.16 | 0.22 | 2.09 | 0.016 | |
| Vaccinate at home vs. health center | -0.64 | -1.08 | -0.21 | 0.004 | 1.27 | 0.79 | 1.75 | <0.001 | |
| Vaccinate at mass site vs. health center | -0.34 | -0.75 | 0.07 | 0.100 | -0.91 | -1.54 | -0.27 | 0.005 | |
| Wait for 1 hr vs. immediate service | -0.55 | -0.90 | -0.20 | 0.002 | -0.71 | -1.24 | -0.18 | 0.009 | |
| Wait for 2 hrs vs. immediate service | -0.78 | -1.12 | -0.44 | <0.001 | 0.48 | -0.13 | 1.10 | 0.126 | |
| Phone vs. online appointment booking | -0.08 | -0.40 | 0.24 | 0.611 | 0.56 | -0.06 | 1.18 | 0.078 | |
| Drop in (no booking) vs. online appointment booking | 0.05 | -0.24 | 0.34 | 0.742 | 0.48 | -0.38 | 1.35 | 0.274 | |
| Vaccinate annually vs. once | -0.70 | -0.92 | -0.48 | <0.001 | 0.86 | 0.64 | 1.09 | <0.001 | |
| Enforcement for air travel vs. no enforcement | -0.64 | -0.93 | -0.34 | <0.001 | -0.55 | -0.93 | -0.17 | 0.005 | |
| Enforcement for work/school vs. no enforcement | -0.97 | -1.39 | -0.56 | <0.001 | -0.79 | -1.33 | -0.25 | 0.004 | |
| Enforcement for recreation vs. no enforcement | -0.77 | -1.08 | -0.45 | <0.001 | 0.38 | 0.02 | 0.73 | 0.038 | |
| A few in the community vaccinated vs. no one | 0.39 | 0.10 | 0.67 | 0.008 | 0.42 | -0.17 | 1.01 | 0.164 | |
| Almost everyone in the community vaccinated vs. no one | 0.58 | 0.29 | 0.86 | <0.001 | 0.40 | -0.22 | 1.02 | 0.201 | |
| Two vaccine doses vs. a single dose | -0.32 | -0.57 | -0.06 | 0.014 | 1.08 | 0.32 | 1.83 | 0.005 | |

S3 Table (e): Weighted mean preferences – Definitely NOT get vaccinated

| Attribute | Relative utilities | | | | Standard deviation | | | |  |
| --- | --- | --- | --- | --- | --- | --- | --- | --- | --- |
|  | Utility | Low CI | High CI | p-value | SD | Low CI | High CI | p-value | |
| Opt-out | -1.57 | -2.51 | -0.63 | 0.001 |  |  |  |  | |
| Vaccinate at pharmacy vs. health center | 0.00 | -0.49 | 0.49 | 0.999 | 1.03 | 0.28 | 1.78 | 0.007 | |
| Vaccinate at community venue vs. health center | -0.11 | -0.58 | 0.37 | 0.651 | -0.81 | -2.15 | 0.52 | 0.234 | |
| Vaccinate at home vs. health center | -0.02 | -0.80 | 0.76 | 0.957 | -1.38 | -1.94 | -0.81 | <0.001 | |
| Vaccinate at mass site vs. health center | 0.20 | -0.27 | 0.67 | 0.399 | -0.22 | -0.91 | 0.47 | 0.527 | |
| Wait for 1 hr vs. immediate service | -0.26 | -0.76 | 0.23 | 0.294 | 0.82 | 0.25 | 1.39 | 0.005 | |
| Wait for 2 hrs vs. immediate service | -0.37 | -0.93 | 0.18 | 0.186 | 1.16 | 0.64 | 1.69 | <0.001 | |
| Phone vs. online appointment booking | -0.09 | -0.53 | 0.35 | 0.696 | -0.72 | -1.35 | -0.09 | 0.025 | |
| Drop in (no booking) vs. online appointment booking | -0.12 | -0.63 | 0.40 | 0.657 | 0.48 | -0.13 | 1.09 | 0.121 | |
| Vaccinate annually vs. once | -0.44 | -0.86 | -0.01 | 0.047 | 0.83 | 0.34 | 1.31 | 0.001 | |
| Enforcement for air travel vs. no enforcement | -0.96 | -1.52 | -0.39 | 0.001 | 1.20 | 0.70 | 1.71 | <0.001 | |
| Enforcement for work/school vs. no enforcement | -0.87 | -1.50 | -0.24 | 0.007 | 1.07 | 0.29 | 1.84 | 0.007 | |
| Enforcement for recreation vs. no enforcement | -1.01 | -1.48 | -0.54 | <0.001 | -0.62 | -1.30 | 0.06 | 0.076 | |
| A few in the community vaccinated vs. no one | -0.07 | -0.41 | 0.27 | 0.681 | 0.40 | -0.14 | 0.94 | 0.149 | |
| Almost everyone in the community vaccinated vs. no one | 0.33 | -0.08 | 0.74 | 0.113 | -0.54 | -1.24 | 0.15 | 0.126 | |
| Two vaccine doses vs. a single dose | -0.38 | -0.70 | -0.05 | 0.022 | 0.86 | 0.48 | 1.24 | <0.001 | |
