# Supplementary material for: Preferences for COVID-19 vaccine distribution strategies in the US: A discrete choice survey
Source: PLoS One. 2021 Aug 20;16(8):e0256394. doi: 10.1371/journal.pone.0256394 (PMC8378751; doi:10.1371/journal.pone.0256394)
Supplement: S4 Table — (DOCX) [file pone.0256394.s004.docx]

**S4 Table (a): Weighted mean preferences – “single dose” latent class group**

| Attribute | Relative utilities | | | | Standard deviation | | | |
| --- | --- | --- | --- | --- | --- | --- | --- | --- |
|  | Utility | Low CI | High CI | p-value | SD | Low CI | High CI | p-value |
| Opt-out | -3.94 | -4.93 | -2.95 | 0.000 |  |  |  |  |
| Vaccinate at pharmacy vs. health center | 0.12 | -0.38 | 0.62 | 0.637 | -0.53 | -1.42 | 0.36 | 0.241 |
| Vaccinate at community venue vs. health center | -0.49 | -0.93 | -0.06 | 0.027 | -0.02 | -0.82 | 0.79 | 0.970 |
| Vaccinate at home vs. health center | -0.98 | -1.62 | -0.34 | 0.003 | 1.21 | 0.56 | 1.85 | 0.000 |
| Vaccinate at mass site vs. health center | -0.12 | -0.73 | 0.49 | 0.701 | 0.73 | -0.21 | 1.68 | 0.129 |
| Wait for 1 hr vs. immediate service | -1.54 | -2.16 | -0.93 | 0.000 | 0.15 | -0.31 | 0.62 | 0.517 |
| Wait for 2 hrs vs. immediate service | -2.42 | -3.10 | -1.75 | 0.000 | -0.70 | -1.43 | 0.03 | 0.060 |
| Phone vs. online appointment booking | 0.48 | 0.01 | 0.95 | 0.045 | 0.77 | -0.17 | 1.71 | 0.110 |
| Drop in (no booking) vs. online appointment booking | -0.19 | -0.56 | 0.19 | 0.326 | 0.53 | -0.07 | 1.13 | 0.083 |
| Vaccinate annually vs. once | 0.14 | -0.26 | 0.54 | 0.505 | 1.01 | 0.43 | 1.59 | 0.001 |
| Enforcement for air travel vs. no enforcement | 0.44 | 0.03 | 0.84 | 0.035 | -0.19 | -1.52 | 1.13 | 0.776 |
| Enforcement for work/school vs. no enforcement | 0.68 | 0.07 | 1.30 | 0.030 | -1.29 | -2.24 | -0.34 | 0.008 |
| Enforcement for recreation vs. no enforcement | 1.24 | 0.60 | 1.89 | 0.000 | 1.24 | 0.36 | 2.11 | 0.006 |
| A few in the community vaccinated vs. no one | 0.65 | 0.23 | 1.08 | 0.003 | -0.46 | -1.05 | 0.13 | 0.126 |
| Almost everyone in the community vaccinated vs. no one | 0.50 | 0.15 | 0.85 | 0.006 | 0.29 | -1.98 | 2.56 | 0.802 |
| Two vaccine doses vs. a single dose | -4.21 | -4.90 | -3.53 | 0.000 | -0.37 | -0.95 | 0.21 | 0.210 |

**S4 Table(b): Weighted mean preferences – “two dose” latent class group**

| Attribute | Relative utilities | | | | Standard deviation | | | |  |
| --- | --- | --- | --- | --- | --- | --- | --- | --- | --- |
|  | Utility | Low CI | High CI | p-value | SD | Low CI | High CI | p-value | |
| Opt-out | -0.45 | -1.03 | 0.13 | 0.127 |  |  |  |  | |
| Vaccinate at pharmacy vs. health center | -0.38 | -0.66 | -0.11 | 0.006 | -0.42 | -0.80 | -0.03 | 0.034 | |
| Vaccinate at community venue vs. health center | -0.18 | -0.54 | 0.18 | 0.322 | 0.83 | 0.39 | 1.26 | 0.000 | |
| Vaccinate at home vs. health center | -1.04 | -1.53 | -0.54 | 0.000 | -0.70 | -1.13 | -0.27 | 0.001 | |
| Vaccinate at mass site vs. health center | -0.67 | -1.01 | -0.32 | 0.000 | -0.44 | -1.04 | 0.15 | 0.143 | |
| Wait for 1 hr vs. immediate service | -0.27 | -0.60 | 0.07 | 0.117 | 0.35 | -0.15 | 0.85 | 0.173 | |
| Wait for 2 hrs vs. immediate service | -0.58 | -0.92 | -0.24 | 0.001 | 0.58 | -0.02 | 1.18 | 0.058 | |
| Phone vs. online appointment booking | 0.44 | 0.20 | 0.69 | 0.000 | 0.83 | 0.41 | 1.24 | 0.000 | |
| Drop in (no booking) vs. online appointment booking | -0.12 | -0.38 | 0.14 | 0.365 | 0.55 | 0.21 | 0.89 | 0.002 | |
| Vaccinate annually vs. once | -0.04 | -0.25 | 0.16 | 0.682 | 0.45 | 0.07 | 0.82 | 0.020 | |
| Enforcement for air travel vs. no enforcement | 0.46 | 0.19 | 0.74 | 0.001 | -0.24 | -1.05 | 0.57 | 0.564 | |
| Enforcement for work/school vs. no enforcement | 0.89 | 0.64 | 1.14 | 0.000 | 0.68 | 0.24 | 1.11 | 0.002 | |
| Enforcement for recreation vs. no enforcement | 0.48 | 0.17 | 0.79 | 0.003 | 0.71 | 0.24 | 1.18 | 0.003 | |
| A few in the community vaccinated vs. no one | 0.40 | 0.18 | 0.61 | 0.000 | 0.28 | -0.13 | 0.70 | 0.178 | |
| Almost everyone in the community vaccinated vs. no one | 0.13 | -0.07 | 0.33 | 0.198 | 0.01 | -0.42 | 0.44 | 0.972 | |
| Two vaccine doses vs. a single dose | 1.72 | 1.49 | 1.95 | 0.000 | 0.72 | 0.42 | 1.02 | 0.000 | |

**S4 Table(c): Weighted mean preferences – “vaccinate once” latent class group**

| Attribute | Relative utilities | | | | Standard deviation | | | |
| --- | --- | --- | --- | --- | --- | --- | --- | --- |
|  | Utility | Low CI | High CI | p-value | SD | Low CI | High CI | p-value |
| Opt-out | -3.34 | -3.84 | -2.83 | 0.000 |  |  |  |  |
| Vaccinate at pharmacy vs. health center | 0.05 | -0.27 | 0.37 | 0.773 | 0.47 | -0.44 | 1.38 | 0.310 |
| Vaccinate at community venue vs. health center | -0.15 | -0.48 | 0.17 | 0.357 | 0.71 | 0.10 | 1.32 | 0.023 |
| Vaccinate at home vs. health center | -0.37 | -0.85 | 0.12 | 0.136 | 1.33 | 0.88 | 1.78 | 0.000 |
| Vaccinate at mass site vs. health center | -0.47 | -0.81 | -0.14 | 0.005 | 0.96 | 0.51 | 1.41 | 0.000 |
| Wait for 1 hr vs. immediate service | -0.72 | -1.05 | -0.38 | 0.000 | 0.53 | 0.11 | 0.95 | 0.014 |
| Wait for 2 hrs vs. immediate service | -1.02 | -1.31 | -0.72 | 0.000 | 0.93 | 0.54 | 1.33 | 0.000 |
| Phone vs. online appointment booking | 0.27 | 0.02 | 0.53 | 0.037 | 0.96 | 0.55 | 1.36 | 0.000 |
| Drop in (no booking) vs. online appointment booking | -0.02 | -0.28 | 0.25 | 0.908 | 1.02 | 0.59 | 1.45 | 0.000 |
| Vaccinate annually vs. once | -3.88 | -4.18 | -3.57 | 0.000 | 0.18 | -0.12 | 0.48 | 0.245 |
| Enforcement for air travel vs. no enforcement | -0.15 | -0.41 | 0.12 | 0.270 | 0.66 | 0.25 | 1.07 | 0.002 |
| Enforcement for work/school vs. no enforcement | -0.50 | -0.79 | -0.21 | 0.001 | 0.53 | 0.18 | 0.87 | 0.003 |
| Enforcement for recreation vs. no enforcement | -0.34 | -0.63 | -0.05 | 0.022 | 0.77 | 0.23 | 1.31 | 0.005 |
| A few in the community vaccinated vs. no one | 0.50 | 0.32 | 0.68 | 0.000 | 0.38 | -0.17 | 0.93 | 0.172 |
| Almost everyone in the community vaccinated vs. no one | 0.69 | 0.49 | 0.89 | 0.000 | 0.83 | 0.46 | 1.19 | 0.000 |
| Two vaccine doses vs. a single dose | -0.70 | -0.89 | -0.51 | 0.000 | 0.68 | 0.05 | 1.32 | 0.034 |

**S4 Table(d): Weighted mean preferences – “vaccination service features” latent class group**

| Attribute | Relative utilities | | | | Standard deviation | | | |  |
| --- | --- | --- | --- | --- | --- | --- | --- | --- | --- |
|  | Utility | Low CI | High CI | p-value | SD | Low CI | High CI | p-value | |
| Opt-out | -3.87 | -4.71 | -3.02 | 0.000 |  |  |  |  | |
| Vaccinate at pharmacy vs. health center | 0.02 | -0.50 | 0.54 | 0.937 | -1.07 | -2.05 | -0.09 | 0.032 | |
| Vaccinate at community venue vs. health center | 0.00 | -0.39 | 0.39 | 0.999 | 1.23 | 0.55 | 1.91 | 0.000 | |
| Vaccinate at home vs. health center | 0.72 | -0.10 | 1.53 | 0.085 | 1.25 | 0.36 | 2.13 | 0.006 | |
| Vaccinate at mass site vs. health center | -1.87 | -2.55 | -1.19 | 0.000 | -0.62 | -1.79 | 0.56 | 0.304 | |
| Wait for 1 hr vs. immediate service | -2.21 | -2.75 | -1.67 | 0.000 | 0.95 | 0.58 | 1.32 | 0.000 | |
| Wait for 2 hrs vs. immediate service | -4.51 | -5.21 | -3.80 | 0.000 | 0.30 | -0.12 | 0.73 | 0.164 | |
| Phone vs. online appointment booking | -0.18 | -0.53 | 0.18 | 0.328 | 0.68 | 0.28 | 1.08 | 0.001 | |
| Drop in (no booking) vs. online appointment booking | 0.45 | 0.08 | 0.81 | 0.017 | -0.75 | -1.19 | -0.31 | 0.001 | |
| Vaccinate annually vs. once | -0.16 | -0.52 | 0.19 | 0.369 | 0.87 | 0.40 | 1.34 | 0.000 | |
| Enforcement for air travel vs. no enforcement | -0.49 | -0.94 | -0.04 | 0.034 | 0.67 | 0.00 | 1.34 | 0.051 | |
| Enforcement for work/school vs. no enforcement | -0.59 | -1.02 | -0.16 | 0.007 | 0.76 | 0.29 | 1.23 | 0.002 | |
| Enforcement for recreation vs. no enforcement | 0.10 | -0.29 | 0.49 | 0.619 | 0.56 | 0.12 | 1.01 | 0.013 | |
| A few in the community vaccinated vs. no one | 0.60 | 0.27 | 0.93 | 0.000 | 0.78 | 0.23 | 1.33 | 0.005 | |
| Almost everyone in the community vaccinated vs. no one | 0.70 | 0.35 | 1.05 | 0.000 | 1.06 | 0.61 | 1.51 | 0.000 | |
| Two vaccine doses vs. a single dose | -0.27 | -0.55 | 0.01 | 0.063 | 0.89 | 0.57 | 1.21 | 0.000 | |

**S4 Table(e): Weighted mean preferences – “social proof” latent class group**

| Attribute | Relative utilities | | | | Standard deviation | | | |  |
| --- | --- | --- | --- | --- | --- | --- | --- | --- | --- |
|  | Utility | Low CI | High CI | p-value | SD | Low CI | High CI | p-value | |
| Opt-out | 0.77 | 0.24 | 1.30 | 0.005 |  |  |  |  | |
| Vaccinate at pharmacy vs. health center | -0.11 | -0.45 | 0.22 | 0.509 | 0.86 | 0.12 | 1.60 | 0.022 | |
| Vaccinate at community venue vs. health center | 0.03 | -0.36 | 0.42 | 0.882 | 1.01 | 0.47 | 1.56 | <0.001 | |
| Vaccinate at home vs. health center | -0.39 | -0.89 | 0.11 | 0.123 | 0.49 | -1.19 | 2.17 | 0.567 | |
| Vaccinate at mass site vs. health center | -0.74 | -1.10 | -0.38 | <0.001 | -0.51 | -1.26 | 0.23 | 0.177 | |
| Wait for 1 hr vs. immediate service | 0.17 | -0.18 | 0.53 | 0.343 | 0.94 | 0.47 | 1.42 | <0.001 | |
| Wait for 2 hrs vs. immediate service | -0.11 | -0.42 | 0.21 | 0.506 | -0.61 | -1.09 | -0.14 | 0.011 | |
| Phone vs. online appointment booking | 0.10 | -0.13 | 0.33 | 0.396 | 0.86 | 0.49 | 1.22 | <0.001 | |
| Drop in (no booking) vs. online appointment booking | 0.55 | 0.28 | 0.81 | <0.001 | 0.81 | 0.41 | 1.22 | <0.001 | |
| Vaccinate annually vs. once | -0.41 | -0.58 | -0.25 | <0.001 | 0.48 | 0.01 | 0.95 | 0.044 | |
| Enforcement for air travel vs. no enforcement | 0.65 | 0.37 | 0.93 | <0.001 | 0.69 | 0.18 | 1.21 | 0.008 | |
| Enforcement for work/school vs. no enforcement | 0.61 | 0.31 | 0.90 | <0.001 | 0.36 | -0.50 | 1.21 | 0.411 | |
| Enforcement for recreation vs. no enforcement | 0.85 | 0.56 | 1.14 | <0.001 | 0.34 | -0.39 | 1.07 | 0.364 | |
| A few in the community vaccinated vs. no one | 2.64 | 2.26 | 3.02 | <0.001 | 0.78 | 0.40 | 1.17 | <0.001 | |
| Almost everyone in the community vaccinated vs. no one | 3.73 | 3.31 | 4.15 | <0.001 | -0.75 | -1.16 | -0.33 | <0.001 | |
| Two vaccine doses vs. a single dose | -0.47 | -0.66 | -0.27 | <0.001 | -0.10 | -1.08 | 0.88 | 0.840 | |

**S4 Table(f): Weighted mean preferences – “indifferent” latent class group**

| Attribute | Relative utilities | | | | Standard deviation | | | |  |
| --- | --- | --- | --- | --- | --- | --- | --- | --- | --- |
|  | Utility | Low CI | High CI | p-value | SD | Low CI | High CI | p-value | |
| Opt-out | -1.81 | -2.14 | -1.47 | 0.000 |  |  |  |  | |
| Vaccinate at pharmacy vs. health center | 0.34 | 0.15 | 0.53 | 0.000 | 0.87 | 0.50 | 1.23 | 0.000 | |
| Vaccinate at community venue vs. health center | -0.06 | -0.22 | 0.09 | 0.431 | 0.51 | -0.08 | 1.10 | 0.092 | |
| Vaccinate at home vs. health center | 0.24 | -0.04 | 0.52 | 0.088 | -0.73 | -1.03 | -0.43 | 0.000 | |
| Vaccinate at mass site vs. health center | 0.04 | -0.13 | 0.20 | 0.657 | 0.68 | 0.28 | 1.08 | 0.001 | |
| Wait for 1 hr vs. immediate service | 0.15 | -0.02 | 0.32 | 0.091 | 0.67 | 0.46 | 0.89 | 0.000 | |
| Wait for 2 hrs vs. immediate service | -0.03 | -0.20 | 0.13 | 0.683 | 0.62 | 0.28 | 0.96 | 0.000 | |
| Phone vs. online appointment booking | -0.49 | -0.64 | -0.34 | 0.000 | 0.26 | -0.89 | 1.40 | 0.659 | |
| Drop in (no booking) vs. online appointment booking | -0.10 | -0.24 | 0.05 | 0.201 | 0.47 | 0.09 | 0.86 | 0.016 | |
| Vaccinate annually vs. once | -0.15 | -0.26 | -0.04 | 0.010 | 0.40 | 0.20 | 0.59 | 0.000 | |
| Enforcement for air travel vs. no enforcement | -0.69 | -0.86 | -0.52 | 0.000 | 0.82 | 0.61 | 1.03 | 0.000 | |
| Enforcement for work/school vs. no enforcement | -0.86 | -1.03 | -0.69 | 0.000 | -0.47 | -0.94 | 0.01 | 0.053 | |
| Enforcement for recreation vs. no enforcement | -0.55 | -0.71 | -0.39 | 0.000 | 0.62 | 0.38 | 0.86 | 0.000 | |
| A few in the community vaccinated vs. no one | -0.13 | -0.26 | -0.01 | 0.035 | 0.29 | -0.17 | 0.75 | 0.221 | |
| Almost everyone in the community vaccinated vs. no one | -0.07 | -0.20 | 0.06 | 0.297 | 0.19 | -0.48 | 0.86 | 0.582 | |
| Two vaccine doses vs. a single dose | -0.33 | -0.43 | -0.24 | 0.000 | 0.46 | 0.19 | 0.74 | 0.001 | |
