# Supplementary material for: Preferences for COVID-19 vaccine distribution strategies in the US: A discrete choice survey
Source: PLoS One. 2021 Aug 20;16(8):e0256394. doi: 10.1371/journal.pone.0256394 (PMC8378751; doi:10.1371/journal.pone.0256394)
Supplement: S5 Table — (DOCX) [file pone.0256394.s005.docx]

|  | Indifferent | | | Immediate service | | | Vaccine features | | | Social proof | | |
| --- | --- | --- | --- | --- | --- | --- | --- | --- | --- | --- | --- | --- |
| Characteristic | Marginal  Probability | Low CI | High CI | Marginal  Probability | Low CI | High CI | Marginal  Probability | Low CI | High CI | Marginal  Probability | Low CI | High CI |
| Age 18-24 yrs | 35.2% | 26.2% | 44.3% | 9.5% | 3.1% | 15.8% | 37.7% | 27.3% | 48.0% | 17.6% | 10.5% | 24.7% |
| Age 25-34 yrs | 42.7% | 34.1% | 51.3% | 4.6% | 1.5% | 7.6% | 39.5% | 31.6% | 47.4% | 13.3% | 8.7% | 17.9% |
| Age 35-44 yrs | 41.4% | 35.3% | 47.4% | 8.9% | 4.5% | 13.4% | 37.7% | 31.6% | 43.9% | 12.0% | 8.3% | 15.7% |
| Age 45-54 yrs | 30.9% | 24.7% | 37.2% | 5.9% | 3.5% | 8.3% | 49.0% | 41.8% | 56.3% | 14.1% | 9.6% | 18.7% |
| Age 55-64 yrs | 29.3% | 24.5% | 34.0% | 9.7% | 7.2% | 12.3% | 48.6% | 43.4% | 53.9% | 12.3% | 9.0% | 15.7% |
| Age 65+ yrs | 20.1% | 15.8% | 24.4% | 9.9% | 6.4% | 13.4% | 60.4% | 54.8% | 66.0% | 9.6% | 6.4% | 12.8% |
| Black/ African American | 38.4% | 32.9% | 44.0% | 6.0% | 3.6% | 8.3% | 47.7% | 41.8% | 53.5% | 7.9% | 5.4% | 10.5% |
| White | 31.5% | 28.3% | 34.6% | 8.5% | 6.6% | 10.3% | 46.9% | 43.6% | 50.2% | 13.1% | 11.1% | 15.2% |
| Asian | 25.5% | 17.7% | 33.3% | 9.7% | 4.3% | 15.0% | 49.3% | 39.8% | 58.8% | 15.5% | 9.3% | 21.7% |
| Other race | 43.5% | 31.3% | 55.7% | 5.4% | 0.0% | 11.5% | 37.8% | 25.5% | 50.1% | 13.3% | 3.3% | 23.3% |
| Democrat | 31.8% | 27.9% | 35.6% | 8.3% | 6.1% | 10.5% | 44.7% | 40.8% | 48.6% | 15.2% | 12.2% | 18.2% |
| Republican | 33.7% | 28.4% | 39.0% | 7.1% | 5.0% | 9.1% | 51.7% | 45.8% | 57.7% | 7.5% | 5.2% | 9.8% |
| Other political group | 32.7% | 27.7% | 37.7% | 8.9% | 5.3% | 12.6% | 45.5% | 40.0% | 51.0% | 12.9% | 9.6% | 16.1% |
| Already/definitely vaccinate | 29.1% | 26.0% | 32.2% | 9.0% | 7.2% | 10.9% | 48.6% | 45.2% | 52.0% | 13.3% | 11.2% | 15.3% |
| Probably vaccinate | 39.0% | 31.7% | 46.4% | 5.3% | 2.7% | 8.0% | 45.1% | 37.5% | 52.8% | 10.5% | 6.6% | 14.4% |
| Probably not vaccinate | 40.0% | 31.2% | 48.9% | 6.0% | 2.7% | 9.4% | 38.9% | 30.0% | 47.7% | 15.1% | 8.0% | 22.1% |
| Definitely not vaccinate | 52.1% | 40.0% | 64.3% | 4.6% | 0.4% | 8.7% | 38.7% | 26.6% | 50.9% | 4.6% | 0.4% | 8.7% |

**S5 Table: Marginal probability of latent class group membership**
