# Supplementary material for: Preferences for COVID-19 vaccine distribution strategies in the US: A discrete choice survey
Source: PLoS One. 2021 Aug 20;16(8):e0256394. doi: 10.1371/journal.pone.0256394 (PMC8378751; doi:10.1371/journal.pone.0256394)
Supplement: S2 Appendix — (DOCX) [file pone.0256394.s007.docx]

Choice experiment modelling is based on random utility theory (RUT) which assumes that the utility (U) for individual i conditional on choice j consists of an explainable component (Vij) and a random component (e_ij_) (formula 1). The random component may capture any combination of unobserved attributes, unobserved preference variation, specification error, measurement error and inherent variability within and between individuals (37).

1. *U_ij =_V_ij_ + e_ij_*

For this analysis we applied dummy coding and created an alternative specific constant to represent the opt-out choice scenario (38, 39). For main effects we conducted mixed logit regression models to account for preference heterogeneity with all attributes included as random parameters and the opt-out ASC included as a fixed parameter. The explainable component (V_ij_) for this experiment is denoted in formula 2 below, where b_1-15_ represents the coefficient for the corresponding attribute level and b_o_ represents the utility associated with the ASC. The baseline attribute category for each attribute is omitted from formulae and estimations, as this attribute has by definition a utility of 0 when dummy coding is used.

1. *V_ij =_ b_o_ + b_1_ waiting time: 1hr + b_2_ waiting time: 2hrs + b_3_ scheduling: phone + b_4_ scheduling: drop-in + b_5_ frequency: annual + b_6_ enforcement: air travel + b_7_ enforcement: work/school +b_8_ enforcement: recreation + b_9_ community vaccinated: few + b_10_ community vaccinated: most + b_11_ dosage: two + b_12_ location: pharmacy + b_13_ location: community pop-up + b_14_ location: home + b_15_ location: mass site*

In this analysis the utility for the ASC includes the utility for the opt-out choice, inattention, experiment complexity and is confounded by the utility estimates for the baseline levels of attributes, b_o_ can therefore not be directly interpreted from model outputs. For this choice experiment our interest was relative utilities and no estimation of preference shares or probability of uptake, we therefore used dummy coded datasets in estimations (40). Mixed logit models were fit using Stata’s mixlogit command which uses simulated maximum likelihood estimators and generates mean utilities for the population and standard deviations of the random coefficients (41).
